# C-terminus-outward orientation of SARS-CoV-2 envelope proteins on viral capsid enables a novel virus–cell interaction pathway

**DOI:** 10.3389/fcimb.2026.1776252

**Published:** 2026-03-12

**Authors:** Jie Xu, Wei Zhao, Yuanyuan Li, Jing Zheng, Yulian Wang, Huimin Sun, Sijin Wu, Baoqing Fu, Yiqiang Wang

**Affiliations:** 1 Eye Institute of Xiamen University, School of Medicine, Xiamen University, Xiamen, China; 2 Department of Laboratory Examination, People’s Hospital of Rizhao City, Rizhao, China; 3 Xiamen Center for Disease Control and Prevention, Xiamen, China; 4 Xiang’an Hospital of Xiamen University, Xiamen University, Xiamen, China; 5 Wisdom Lake Academy of Pharmacy, Xi’an Jiaotong-Liverpool University, Suzhou, China

**Keywords:** COVID-19, envelope protein, microbe-host interaction, receptor, SARS-CoV-2

## Abstract

Since the onset of COVID-19, emphasis has been largely placed on spike proteins, while other molecules of severe acute respiratory syndrome coronavirus 2 (SARS-CoV-2), such as the 78-aa-long envelope protein (E-pr), have been largely overlooked. This study was conducted to identify the orientation of E-pr in the envelope of the living SARS-CoV-2 virus and to confirm whether the viroid E-pr plays a role in the attachment and stimulation of the host cells. Using a customized antisera against the C-terminal half of E-pr (EC_38_, 38 aa), we demonstrated that the EC_38_ segment was located outside of the intact virus. Synthetic peptides with the sequence of the EC_38_ segment were bound to and modulated gene expression in cultured human vascular endothelial cells (HUVECs), and altered genes were enriched for the ontology terms “hemostasis” and “regulation of systemic arterial blood pressure by hormone.” EC_38_-mediated pull-down of HUVEC membrane proteins plus mass spectrometry revealed that the EC_38_-interacting proteins were enriched for “actin cytoskeleton organization,” “cell–cell interaction,” “cell–cell adhesion,” “hemostasis,” “viral infection pathways,” “cytokine signaling in immune system,” etc., among which CKAP4, vimentin, and ATP5B were further identified to bind EC_38_ peptides. Molecular docking supported the potential binding of the E-pr with the ATP synthase. In summary, by displaying a C-terminal-outward orientation on the surface of the SARS-CoV-2 virus, E-pr was able to adhere to and stimulate host cells. Future studies should address whether the E-pr pathway could be utilized as a target for managing SARS-CoV-2 infection in humans.

## 1 Introduction

The worldwide coronavirus disease 2019 (COVID-19) pandemic, which began at the end of 2019, caused approximately 7.1 million deaths (as of 26 June 2025), along with approximately two-fold excess mortality. At the beginning of the pandemic, much of the knowledge supporting disease management was adopted directly from earlier studies on severe acute respiratory syndrome coronavirus 1 (SARS-CoV-1) or other members of *Orthocoronavirinae* or *Coronaviridae*. Later, more knowledge on severe acute respiratory syndrome coronavirus 2 (SARS-CoV-2) per se was accumulated in both basic biomedical aspects and clinical aspects. Among the three viral membrane-anchored structural proteins, the spike (S-) proteins became the sole target for pathogenesis studies or for developing vaccines or antibody-based therapeutics, presumably due to their larger size and outermost location at the viral particle surface (Bar-On et al., 2020). The other two smaller membrane-associated structural proteins, i.e., the membrane protein (M-pr) and the envelope protein (E-pr), were overlooked in this aspect, but they were recognized to be of no less significance for the pathogenicity of the virus. At the molecular level, E-pr was proposed to form a pentameric viroporin and participate in the regulation of the virus life cycle in host cells, especially in the endoplasmic reticulum-Golgi apparatus intermediate compartment (ERGIC)/Golgi membranes (Mandala et al., 2020; Cao et al., 2021). While it is well established that the full E-protein sequence comprises an N-terminal domain, a transmembrane domain, and a C-terminal domain, the orientation of the E-protein viroporin within organelle membranes remains controversial (Nieto-Torres et al., 2011; Schoeman and Fielding, 2019; Mukherjee et al., 2020). In more detail, Nieto-Torres et al. used a stringent immunolabeling method to demonstrate that E-pr in membranes of intracellular organelles took an N_lumen_–C_cytoplasmin_ orientation (Nieto-Torres et al., 2011). However, using an *in vitro* translation system in the presence of ER microsome and with the isotope tracing method, Duart et al. discovered that different coronaviruses in cells might manifest robust topologies of E-pr, depending on the actual amino acid sequences in different variants (Duart et al., 2021). In early 2006, Yuan et al. proposed that the E-pr of the same virus strain demonstrated mixed topologies in organelle membrane, whereby in some cytoplasmic compartments, some E-pr crossed the lipid membrane with the C-terminus (Ct-) outside and the Nt inside the compartment, while some other E-pr took reverse directions, or even presented a U-shape transmembrane domain with both termini outward (Yuan et al., 2006). Regretfully, all the above studies were performed in cell-derived membranes or artificial bilayer lipid membranes (Khattari et al., 2006; Verdia-Baguena et al., 2012) but not in mature virus per se. Several recent studies have addressed how host cells sense soluble E-pr (either naturally derived from COVID-19 patients’ sera or recombinant products) (Tang et al., 2023; Grant et al., 2025). However, it was more desirable to understand how E-pr is presented in natural live virus, since the orientation of E-pr in viral capsids will determine which part of E-pr is exposed outside of the viral capsid and thus further contribute to the features of the virus–host interface (e.g., attachment, recognition, taking effects, etc.). Here, we report for the first time that the C-terminal half of E-pr (38 aa-long, hereafter EC_38_, RLCAYCCNIVNVSLVKPSFYVYSRVKNLNSSRVPDLLV) was outside of the envelope in mature virus, and the synthetic EC_38_ peptides were both immunogenic and antigenic *in vivo*. Potential partners in human endothelial cells that interacted with EC_38_ peptides were also identified.

## 2 Materials and methods

### 2.1 Research design

This preliminary study was conducted to confirm, first, how the E-pr is oriented in the SARS-CoV-2 capsid and, second, whether the external part of E-pr exposed in virus surface (e.g., the EC_38_ part, as first proposed and subsequently defined; refer to **Supplementary Information** for details) is structurally and/or functionally involved in virus–host cell interaction in a living system. Understandably, the use of specific antibodies against the studied segments would be helpful. However, due to the lack of commercially available antibodies against the EC_38_ segment of interest, a homemade anti-EC_38_ serum was generated by immunizing C57BL/6 mice with synthetic EC_38_ peptides, and this antisera was used to detect natural full-length E-pr. To examine whether the EC_38_ segment in the intact virus was located outside or inside the viral capsid, two strategies were utilized. In the first strategy, inactivated SARS-CoV-2 virus originally generated from Vero cells were attached to fixed 293T-ACE2hR cells (a gift from Dr. Quan Yuan; Zhang et al., 2021). The virus–cell aggregates were divided into two groups, with one group permeabilized with Triton X-100 to allow antigens within the virus and cells to be accessible by antibodies; otherwise, the antibodies would only detect the antigen on the outer surface of the cell–virus complex. In the second strategy, anti-EC_38_ and anti-MN_19_ antisera were conjugated to Protein A agarose and incubated with live or inactivated COVID-19 viruses. Bound viruses were detected by reverse transcription-PCR using an appropriate assay kit as detailed in the **Supplementary Methods**. For parallel comparison in some settings, the documented external segment of M-pr (i.e., MN_19_, MADSNGTITVEELKKLLEQ, corresponding to the 19-aa-long N-terminal segment of M-pr; Gonzalez-Orozco et al., 2024) was utilized along with EC_38_.

In functional studies, cultured human umbilical vein endothelial cells (HUVECs, China Center for Type Culture Collection, Wuhan, China) were used to model the vascular endothelium. Briefly, after treatment of HUVECs with EC_38_ peptides, RNA-seq was used to measure the transcriptome changes of HUVECs, while pull-down assays coupled with mass spectrometry were used to detect the interactome of the EC_38_ peptides on HUVEC cellular membranes. Bioinformatic analyses were performed using adequate programs to analyze the two cohort data (i.e., transcriptome and interactome) to build up the interacting scheme for the involvement of E-pr in COVID-19 pathogenesis related to the vascular endothelial function.

Except for key materials and methods discussed below, other methods are available in the **Supplementary Information**.

### 2.2 Immunization and obtainment of antibodies from animals against E- or M-proteins

Experiments involving animal use were approved by the Animal Welfare and Ethics Committee of Xiamen University (File# XMULAC20201004) and were conducted at the Animal Center of Xiamen University with institutional guidelines. Specific pathogen-free C57BL/6 male mice (6–8 weeks old) were used for immunogenicity studies and anti-serum production (**Supplementary Methods**), which were subsequently utilized for labeling studies.

### 2.3 Measurement of antibodies in the sera of human subjects against SARS-CoV-2 antigens

The use of human blood samples from recovered COVID-19 patients or healthy donors was approved by the Institutional Review Board (IEC-016-6.1/MR-77-01) of Rizhao People’s Hospital, and the principles of the Declaration of Helsinki were observed. Informed consent was obtained from the patients. For recruitment of participants, diagnosis of COVID-19 cases was conducted using the real-time reverse transcription-polymerase chain reaction (RT-PCR) assay according to clinical procedures with a commercial kit (DA0990, DaAn Gene Co., Ltd., Guangzhou, China). A negative RT-PCR result, characterized by cycle threshold (Ct) values for the N gene and ORF1ab region exceeding 40, indicated a non-infection. For sera preparation, 2 mL of peripheral blood sample was drawn from each participant and allowed to sit for half an hour at room temperature before centrifugation at 4,000 rpm for 5 min, and the serum was collected and stored at −80°C. The presence or level of antibodies against E-, M-, or S-proteins was measured using routine ELISA, as detailed in the **Supplementary Information**.

### 2.4 Preparation of live SARS-CoV-2 virus

Live SARS-CoV-2 virus was isolated, expanded, and used in the BSL-3 Lab of Xiamen Center for Disease Control as previously reported (Zhang et al., 2022) in accordance with institutional biosafety rules, with approval from the institutional review board (Serial No. XJK/LLSC(2020)003). In brief, the virus was prepared in VERO-E6 cells, harvested, titered, and resuspended to 2 × 10^7^ PFU/mL in PBS with 1% fetal bovine serum for experimental use.

### 2.5 Labeling and visualization of EC_38_ segments and N-proteins in SARS-CoV-2 viroids anchored to cells

**Figure 1:**
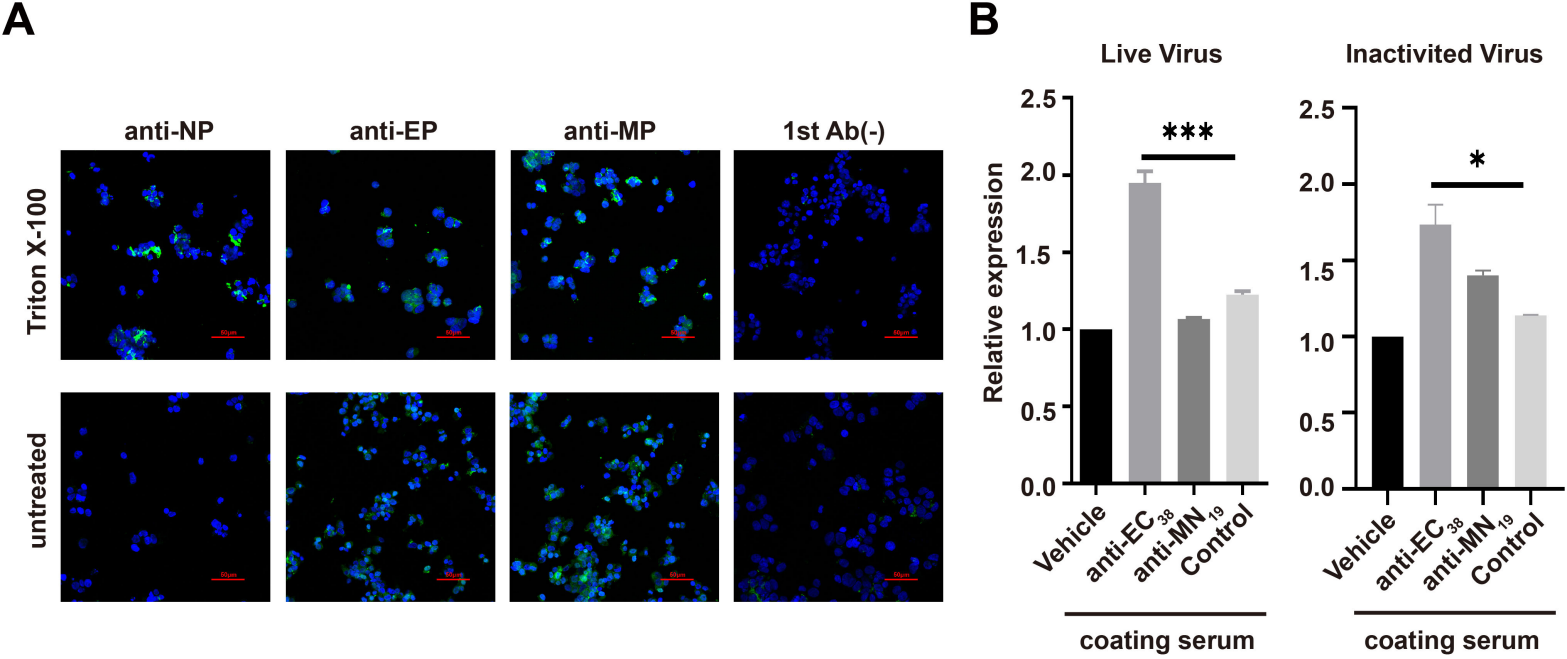
Orientation of E-protein on virus envelope as detected via affinity immunofluorescence or binding onto cells or beads. **(A)** Nucleocapsid proteins located within lipid membranes can only be revealed with an antibody (green) after permeabilization, whereas anti-E antisera can reveal the virus without permeabilization. Imaging setup: channel 1 (DAPI): laser wavelength: 405.0, laser power: 13.0%, PMT HV: 99, PMT offset: 0. Channel 2 (Alexa Fluor 488): laser wavelength: 488.0, laser power: 9.0%, PMT HV: 66, PMT offset: 0. **(B)** Sera from mice challenged with EC_38_ or MN_19_ peptides, or administered PBS (control group), were coupled to the surface of Protein A agarose beads, and the resulting beads were used for capturing live or inactivated SARS-CoV-2 virus, which was subsequently measured using the RT-PCR assay. “Vehicle” refers to substituting sera with PBS during the coating of beads. *N* = 3 for the replicates of the samples. *p < 0.05, **p < 0.01, ***p < 0.001, ****p < 0.0001, vs. control, all through Student’s *t*-test.

To examine whether the EC_38_ segment was outside the viral capsid or inside, two strategies were utilized. In the first strategy, the inactivated SARS-CoV-2 virus originally generated from Vero cells (Sinovac Life Sciences Co., Ltd., Beijing, China) was attached to fixed 293T-ACE2hR cells. In brief, HEK 293T-hACE2 cells grown at the exponential phase were fixed in 4% (w/v) paraformaldehyde (Biosharp, Hefei, China) for 30 min on ice. Cells were washed with PBS three times to completely remove residual paraformaldehyde. Fixed cells were incubated with inactivated SARS-CoV-2 virus at a 1/1,000 ratio for 1 h at room temperature. After centrifugation at 300*g* to remove unbound virus, the residual virus–cell aggregates were divided into two groups, with one group permeabilized with 0.2% Triton X-100 for 30 min to allow antigens inside the virus or cells to be accessible by their antibodies added to the aggregates; otherwise, only the antigens on the outer surface of the cell–virus complex would be detectable by antibodies. After washing with PBS three times, cells were incubated at room temperature for 1 h with primary commercial antibody against N-protein (Cat# DA027, Novoprotein Company, Suzhou, China) at 2 μg/mL, or with homemade normal mouse serum, anti-EC_38_, or anti-MN_19_ antiserum prepared as above. After washing with PBS three times, cells were incubated with Alexa Fluor 488-labeled goat anti-mouse IgG antibody (1:500, ab150117, Abcam). The nucleus was counterstained with DAPI (Alexis, Leysin, Switzerland), and fluorescence signals were captured using a Nikon A1R fluorescence confocal microscope (Nikon, Tokyo, Japan), with features provided individually for each figure (as exemplified in the legend of **Figure 1**).

### 2.6 Measurement of SARS-CoV-2 viroids bound to anti-EC_38_ or anti-MN_19_ antisera-coated beads

Anti-EC_38_ and anti-MN_19_ antisera or control sera were diluted to 1:5 in PBS and conjugated to Protein A agarose (AAT Bioquest, CA) via incubation at room temperature for 1 h. Then, the beads were washed three times with PBS, followed by incubation with inactivated SARS-CoV-2 or live viruses. After three washes with PBS, beads (now with bound viruses) were treated like clinical specimens and subjected to reverse transcription-PCR as above. The relative quantity of the bound virus was compared among groups, but the measurement of the absolute quantity of the captured virus was not attempted.

### 2.7 Measurement of binding of EC_38_ or MN_19_ peptides and S-trimer proteins to HUVEC cells

HUVEC cells were grown in 96-well plates to a confluent layer. His-tagged polypeptides (including EC_38_, MN_19_, and S-trimer) were added to each well (50 μL/well) at different concentration gradients. After 60 min, unbound molecules were washed three times with PBS, followed by incubation with 1:10,000 diluted HRP-conjugated Anti-6X His tag^®^ antibody (ab1187, Abcam, Cambridge, MA) for 1 h. After washing three times, TMB solution (Beyotime, Shanghai, China) was added to the plate for 30 min, and the plate was read at 370 nm in a microplate reader.

### 2.8 FACS assay of EC_38_ peptides or their subsegments binding onto HUVEC cells

Single HUVEC cells were harvested during the exponential growth phase. FITC-conjugated EO, EN, EM, EC, and EL peptides were each added to a final concentration of 5 μM, and incubation was continued for 60 min. After washing three times with PBS, HUVECs were resuspended at 1 × 10^7^ cells/mL in PBS containing 5% BSA and were analyzed with a CytoFLEX S Flow Cytometer (Beckman Coulter, Brea, CA). Prior to acquisition, the cell suspension was filtered through a cell strainer. A minimum of 10,000 events was acquired per sample. Cells were analyzed using a 488-nm laser for excitation and a 530-nm filter for emission (FL1 channel) to detect FITC fluorescence. Acquired data were analyzed with FlowJo software (Tree Star, Ashland, OR). The percentage of positive cells and the geometric mean fluorescence intensity (MFI) were recorded for each sample.

### 2.9 Measurement of the effects of EC_38_ peptides on HUVECs via the RNA-seq assay

HUVECs were seeded in 6-well plates and allowed to grow to the exponential phase. EN_38_ and MN_19_ peptides were added to the medium at a final concentration of 5 μM for overnight stimulation. After removing the medium, total RNA was isolated from the cells using TRIzol lysis reagent (Life-iLab Biotech, Shanghai, China). Transcriptome assay was performed using the Illumina single-color Human BeadChip HT12 v4 (Illumina, San Diego, CA) under a contracted service (contract number F21FTSSCKF2904_POEvnagT) from Wuhan Huada Medical Laboratory Co., Ltd. (Wuhan, China). After data normalization and calculation, differentially expressed genes (DEGs) in EN_38_- or MN_19_-treated cells were analyzed using Metascape (Zhou et al., 2019).

### 2.10 Identification of EC_38_ peptides’ interactome in HUVEC via the pull-down–mass spectrometry assay

To retrieve all possible interactors of the EC_38_ segment in HUVEC cells, membrane and cytosol proteins were extracted using a Membrane and Cytosol Protein Extraction Kit (Beyotime) as a starting protein pool. At the same time, 500 μg of His-tag EC_38_ peptides were mixed with 20 μL of BeyoGold™ His-tag Purification Resin (Beyotime) at 4°C for 60 min under gentle shaking. The resin was spin-washed with washing buffer (50 mM of NaH_2_PO_4_, 300 mM of NaCl, pH 8.0) containing 2 mM of imidazole (Shanghai BioScience and Technology Company, Shanghai, China) three times and mixed with 100 μg of HUVEC-derived membrane proteins at 4°C for 60 min under gentle shaking. After three washes as above, bound proteins were eluted with 50 mM imidazole and resolved by SDS-polyacrylamide gel electrophoresis. After routine silver staining with Fast Silver Stain Kit (Beyotime), the gel slice with visible bands was recovered and subjected to routine mass spectrometry. Bruker timsTOF Pro instrument and accompanying software (Billerica, MA) were utilized to extract information for the corresponding proteins, which were further subjected to clustering with DAVID v6.7 (Database for Annotation, Visualization, and Integrated Discovery, NIH, Bethesda, MD) (Sherman et al., 2022). Physical interaction of the interacting proteins of interest (e.g., vimentin, CKAP4, ATP5B) with EC_38_ was confirmed with the co-pull-down and Western blotting assay, as detailed below (for ATP5B) or in the **Supplementary Information**.

### 2.11 Effect of anti-ATP5B antiserum on EC_38_ binding to HUVECs

HUVEC cells were grown in 96-well plates and grew to a single layer. Undiluted anti-ATP5B serum was added to each well (50 μL/well). After 60 min, the plates were washed three times with PBS and then incubated with different concentrations of EC_38_ polypeptides for 60 min at room temperature. The wells were then incubated with HRP-conjugated Anti-6X His tag^®^ antibody (ab1187, Abcam) and diluted at 1:10,000. TMB solution (Beyotime) was added to the plate, and data were measured at 370 nm using a Multiskan GO Spectrophotometer (Model 1510) (Thermo Fisher Scientific, MA).

### 2.12 3D modeling of E-proteins binding onto ATP synthases

We used the E-protein pentamer modeling structure from a previous study (Yang et al., 2022) to investigate the potential interaction modes of E-pr with F1-ATPase (1e79, RCSB). ZDOCK software was used to predict the binding between these two complexes, with no constraints placed on the interfacial residues. The Rosetta score application was used to rescore the docking results. The top 100 docking results with the best performance in Rosetta score and ZDOCK score were selected using PyMOL 2.4.2 (Yuan et al., 2016) (PyMOL Molecular Graphics System, Version 2.0 Schrödinger, LLC) to assess the interaction details.

## 3 Results

### 3.1 The C-terminal half of the SARS-CoV-2 E-protein was immunogenic in humans and mice

**Figure 2:**
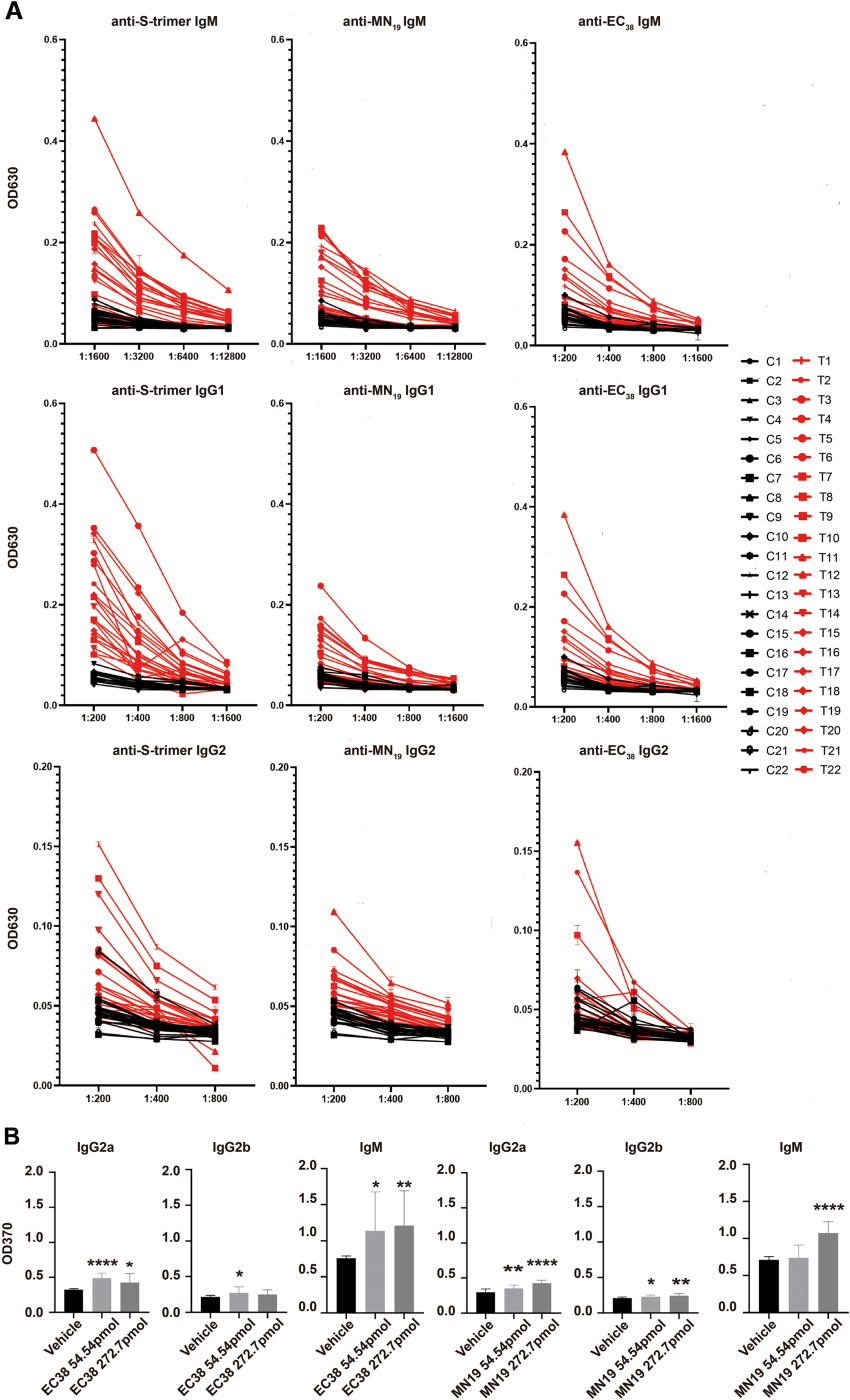
Detection of binding reactivity of human and mouse sera with hypothetical antigens of SARS-CoV-2. **(A)** Sera were collected from recovered COVID-19 patients or from control healthy donors (i.e., with a negative report for RT-PCR detection of SARS-CoV-2 genes). Synthetic peptides (EC_38_ and MN_19_) and recombinant S-trimers were used as absorbing antigens for the following ELISA assay. Please note that comparison of titers of different Ig classes of the same samples should be made with data obtained at the same dilutions. For example, OD_630_ readouts for IgM at a 1:1,600 dilution of samples were significantly higher than those for IgG1, and OD_630_ readouts for IgG1 at 1:800 were higher than those for IgG2. Accurate titration was not attempted. **(B)** Antibody measurement in EC_38_ or MN_19_ peptide-immunized mice. C57BL/6 mice (*n* = 4/group) were immunized with two doses (low: 54.54 pmol, high: 272.7 pmol) of EC_38_ peptides or MN_19_ peptides with CpG-ODN plus incomplete Freund’s adjuvant as adjuvant mixture (20 µg of CpG-ODN 1018 plus 100 µL of IFA) three times on day 0, day 21, and day 42. Antibodies against EC_38_ or MN_19_ peptides were titered with ELISA and compared with control sera. *N* = 4 for each group, and *p < 0.05, vs. vehicle, all through Student’s *t*-test with Prism.

Based on the majority of studies and program predictions (Mandala et al., 2020; Cao et al., 2021; Duart et al., 2021), the 75-aa-long SARS-CoV-2 E-pr was roughly divided into two even halves: a 37-aa-long N-terminal half that could be further divided into an extra-membrane N-terminus and a transmembrane domain and a 38-aa-long C-terminal half (EC_38_). The accurate boundaries among these three segments have not been agreed upon by all, which does not, however, affect the underlying rationale or interpretation. A published study (Jakhar and Gakhar, 2020) and predictions using the Immune Epitope Database (IEDB) (Vita et al., 2019) or BepiPred (Jespersen et al., 2017) suggested that the EC_38_ segment contained B-epitopes (**Supplementary Figure 1**), thus should be immunogenic in the human histocompatibility complex context. In line with these predictions, sera from COVID-19 patients approximately 1 month post-diagnosis were positive for anti-EC_38_ reactivity, as well as for the synthetic immunogenic MN_19_ peptides and recombinant S-proteins (**Figure 2A**). Comparing the optical densities for different Ig classes (IgM vs. IgG1 and IgG1 vs. IgG2) of similar samples demonstrated that the titers of IgM class reactivity against EC_38_ and S-trimer were much higher than those of IgG1 or IgG2, respectively, while the fold difference of these three classes of Ig for MN_19_ was significantly smaller (**Figure 2A**).

To check whether the anti-EC_38_ reactivity in patients’ sera could be due to EC_38_ segment’s immunogenicity or just due to cross-reactivity of the bulk antibodies, EC_38_ and MN_19_ peptides were used to immunize immunocompetent mice with CpG oligonucleotides plus incomplete Freund’s adjuvant as an adjuvant. After the third immunization, and similar to human subjects, the sera from mice demonstrated antibody reaction against EC_38_ and MN_19_ peptides, respectively (**Figure 2B**), confirming that the Ct half of E-pr was immunogenic *in vivo* just as the N-terminus of M-pr (at least in mice).

### 3.2 The E-protein took a C-terminus-outward orientation in SARS-CoV-2 viral envelope

Due to a lack of specific monoclonal anti-EC_38_ antibodies, the immune sera from the EC_38_ peptide-immunized mice were subsequently used to label the EC_38_ segment in the following experiments. Anti-MN_19_ sera were similarly produced and utilized in parallel (**Supplementary Figure 2**).

Obviously, if the EC_38_ segment was located outside the mature SARS-CoV-2 virus, the antibodies induced against this segment in either humans or mice might play protective roles to some extent. On the contrary, if the EC_38_ segment was inside the mature SARS-CoV-2 virus, the antibodies against this segment, even if produced, would not be able to protect against the live virus at all due to their inaccessibility to their targets. To examine whether the EC_38_ segment was outside or inside the viral envelope, two experiments were performed using inactivated or live SARS-CoV-2 virus, respectively. In the first setting, 293T-ACE2hR cells in the exponential phase were used to capture inactivated virus in culture medium and subsequently were permeabilized using Triton X-100 to make antigens in the virus and the cells accessible by antibodies. Unpermeabilized virus–cell conjugates were used as control. After incubation with the customized anti-EC_38_ sera, anti-MN_19_ sera, or commercial anti-nucleocapsid protein (NP) antibodies and following staining with the fluorescence-labeled secondary antibody, the anti-EC_38_ and anti-MN_19_ antibodies produced positive staining signals for both permeabilized and non-permeabilized samples, while the anti-NP antibodies did not stain the unpermeabilized samples positively (**Figure 1A**). In the second experiment, anti-EC_38_ and anti-MN_19_ sera were conjugated to Protein A agarose and incubated with live or inactivated COVID-19 viruses, and bound viruses were detected using reverse transcription-PCR. The results showed that the anti-EC_38_ sera captured both live and inactivated SARS-CoV-2 virus efficiently, but the anti-MN_19_ sera captured the virus only marginally without statistical significance (**Figure 1B**). Taken together, these data demonstrated that the EC_38_ segment of E-pr, just like the N-terminus of M-pr, was located outside the virus envelope and was accessible by exogenous antibodies. On the contrary, NP was within the virus and required membrane permeabilization to be detected by its antibodies.

### 3.3 Exterior segments of E-proteins bound onto human vascular endothelial cells *in vitro*

**Figure 3:**
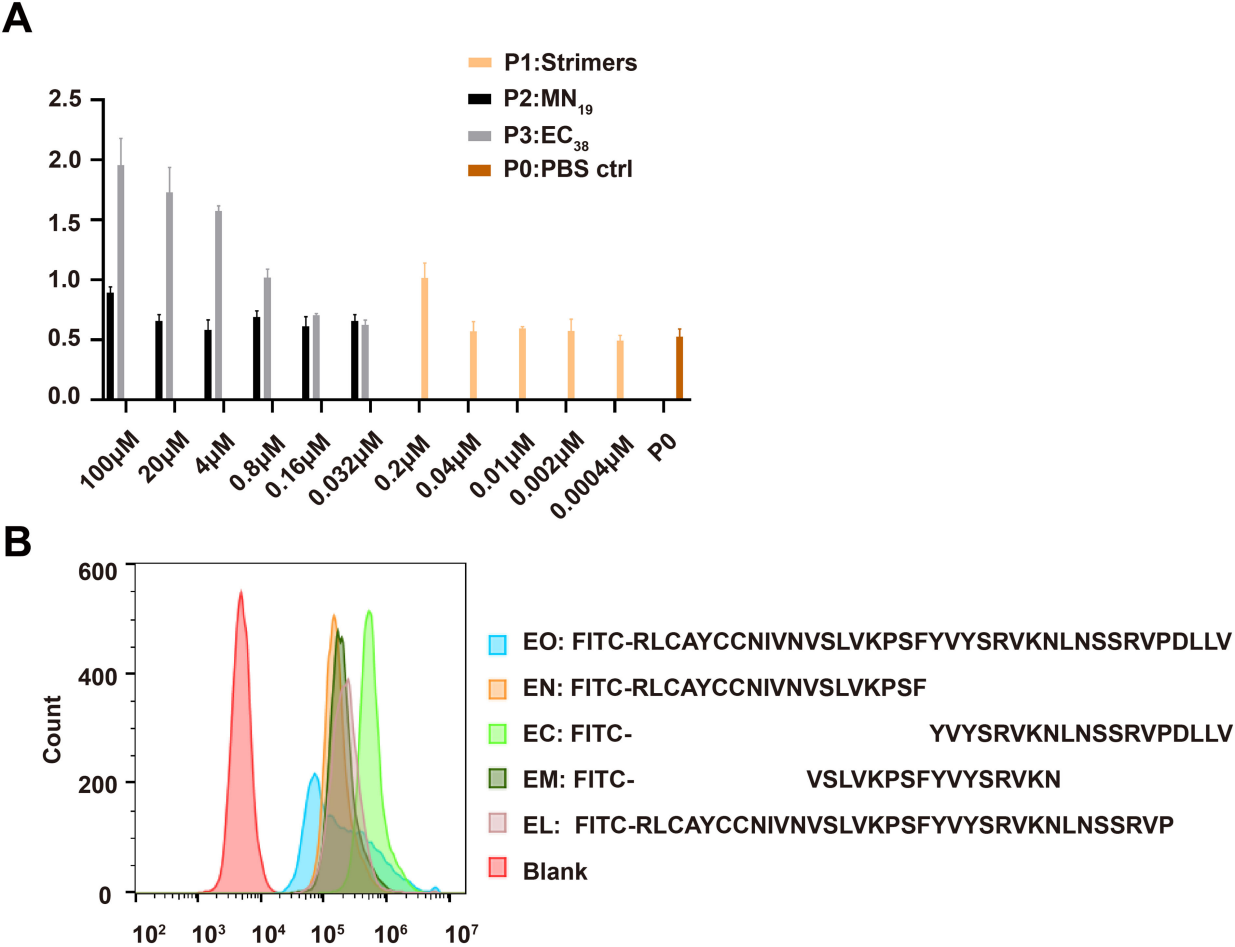
Binding of EC_38_-associated peptides to human vascular endothelial cells. **(A)** ELISA was applied to assess the binding ability of synthetic EC_38_ or MN_19_ peptides to HUVEC, with S-trimer as a positive control. *N* = 3 for each set. Shown were representatives of two repeated experiments with similar results. **(B)** Flow cytometry was used to detect the binding between HUVECs and FITC-labeled EC_38_-associated polypeptide of different lengths. This assay was repeated three times with a similar conclusion.

The above finding that the EC_38_ segment was outside the SARS-CoV-2 virus envelope prompted us to ask if this segment would bind to and impact the biology of the host cells. Bearing in mind the viremia symptom observed in severe COVID-19 patients (Giordo et al., 2021; Liu et al., 2021), cultured HUVECs were used as a cellular model of the circulation system to test the binding of EC_38_ and MN_19_ peptides to HUVECs and the potential consequence in terms of transcriptome. The EC_38_ peptides, but not the MN_19_ peptides, exhibited a dose-dependent binding to HUVEC cells (**Figure 3A**). S-trimer proteins at a comparable molar concentration (0.2 µM) manifested binding comparable to the 0.8-µM EC_38_ peptides (**Figure 3A**). To further explore which part of the EC_38_ might contribute to the interactions between EC_38_ and HUVECs, four shorter peptides representing different parts of EC_38_ (EN, EC, EM, and EL, respectively) were compared using flow cytometry for their binding to HUVECs (**Figure 3B**). Interestingly, while EC_38_ peptides always produced a wider histogram curve along the fluorescence intensity axis, the four shorter peptides produced narrower histogram shapes at different places along the axis. According to the rationale of flow cytometry, it could be interpreted that all cells in the same HUVEC culture preparation manifested diverse binding affinities for EC_38_ peptides, while their binding ability for the four shorter peptides was sequence-dependent and relatively consistent (**Figure 3B**).

### 3.4 Exterior segments of E-proteins (EC_38_) modulated gene expression in human vascular endothelial cells

To check if the binding of peptides to HUVECs was accompanied by functional consequences within the cells, HUVECs were treated with EC_38_ and MN_19_ peptides (5μM) for 24 h, and their transcriptome was measured via RNA sequencing. Data were deposited in the Gene Expression Omnibus as GSE268369. Among the annotated 15,964 genes, 74 genes were classified as upregulated and 66 downregulated by over 1.5-fold (*p* < 0.05) following treatment with EC_38_ peptides (**Supplementary Table 1**). Analysis of the altered genes using Metascape revealed that genes in “hemostasis” and “regulation of systemic arterial blood pressure by hormone” demonstrated top enrichment in regulated genes in the EC_38_ peptide-treated cells (**Table 1**). This finding aligns with and might help explain the coagulation disorders and hypertension recorded in COVID-19 patients (as discussed later). In comparison, only approximately one-sixth of altered genes induced by EC_38_ peptides were also observed following MN_19_ peptide treatment (**Supplementary Table 3**), suggesting that the exterior segments of M-pr and E-pr (i.e., MN_19_ and EC_38_, respectively) did not substantially overlap in their stimulation of HUVECs. A subsequent work therefore focused on EC_38_.

**Table 1:** Top 10 clusters of DEGs induced by EC_38_ with representative enriched terms (one per cluster), as identified by Metascape.

Category	Term	Description	LogP	Symbols	Count
Reactome Gene Sets	R-HSA-109582	Hemostasis	−5.39	ACTN2, CEACAM5, CEACAM8, MPL, P2RY1, SERPINF2, PSG7, PTGIR, H3C1, H3C8, H3C2, KIF21B, DOCK8	13
GO Biological Processes	GO:0001990	Regulation of systemic arterial blood pressure by hormones	−4.76	PCSK5, SERPINF2, CORIN, SUCNR1, CD34, CHRNB2, OPRL1, ID2, P2RY1, MAP2K6, CASP1, BTK, NLGN1	13
KEGG Pathway	hsa05034	Alcoholism	−4.67	H2AC13, H2BC14, H3C1, H3C8, H3C2, SHC2, CALML6, CASP1, CR1, IL1A, MAP2K6, BTK, ST6GALNAC2, FXYD6, ANO1, NOD2, CATSPER1, RUNX2, KIF21B, DOCK8, ID2, HEY1	22
Reactome Gene Sets	R-HSA-388396	GPCR downstream signaling	−4.58	BTK, CCR4, GNAT2, OPRL1, P2RY1, PDE4A, PTGIR, NPFF, PDE8B, GPR83, SUCNR1, ARHGEF37, ACTN2, ANO1, SORCS2, CHRNB2	16
GO Biological Processes	GO:0030855	Epithelial cell differentiation	−3.93	CD34, DNASE1L2, ID2, IL1A, KRT17, KRT34, POU4F3, HEY1, FAM20C, TMC1, SYNE4	11
GO Biological Processes	GO:0051963	Regulation of synapse assembly	−3.79	CHRNB2, NLGN1, FLRT3, RAB17, GPRASP3, CAMKV, COBL, ACTN2, EFNA2, POU4F3, ANO1, GARIN1A	12
GO Biological Processes	GO:0098742	Cell–cell adhesion via plasma-membrane adhesion molecules	−3.77	CEACAM5, CEACAM8, MPZL2, NLGN1, FLRT3, PCDHGA5, PCDHB4, CD34	8
GO Biological Processes	GO:0032663	Regulation of interleukin-2 production	−3.75	CD34, CR1, IL1A, NOD2, BTK, CHRNB2, ID2, CCR4, GNAT2, MPL, NAPSA, RDH12, DOCK8, SIGLEC16, ADAMTS18, P2RY1, SERPINF2, PDE8B, CASP1, CALML6	20
Canonical Pathways	M110	PID IL1 PATHWAY	−3.34	CASP1, IL1A, MAP2K6, CALML6	4
WikiPathways	WP4877	Host–pathogen interaction of human coronaviruses MAPK signaling	−3.27	MAP2K6, IFITM1, IFITM2, APOBEC3F, BTK, CASP1, CR1, NOD2, NLRC5, RNASE7, TMEM45B	11

This was a direct download from the Metascape analysis result page under the “Pathway and Process Enrichment Analysis” block without changing the merit of the contents. As stated in the program, “for each given gene list, pathway and process enrichment analysis have been carried out with the following ontology sources: KEGG Pathway, GO Biological Processes, Reactome Gene Sets, Canonical Pathways, CORUM, WikiPathways, and PANTHER Pathway.”.

### 3.5 A variety of membrane-associated proteins in HUVECs interacted with the exterior segment of EC_38_

**Figure 4:**
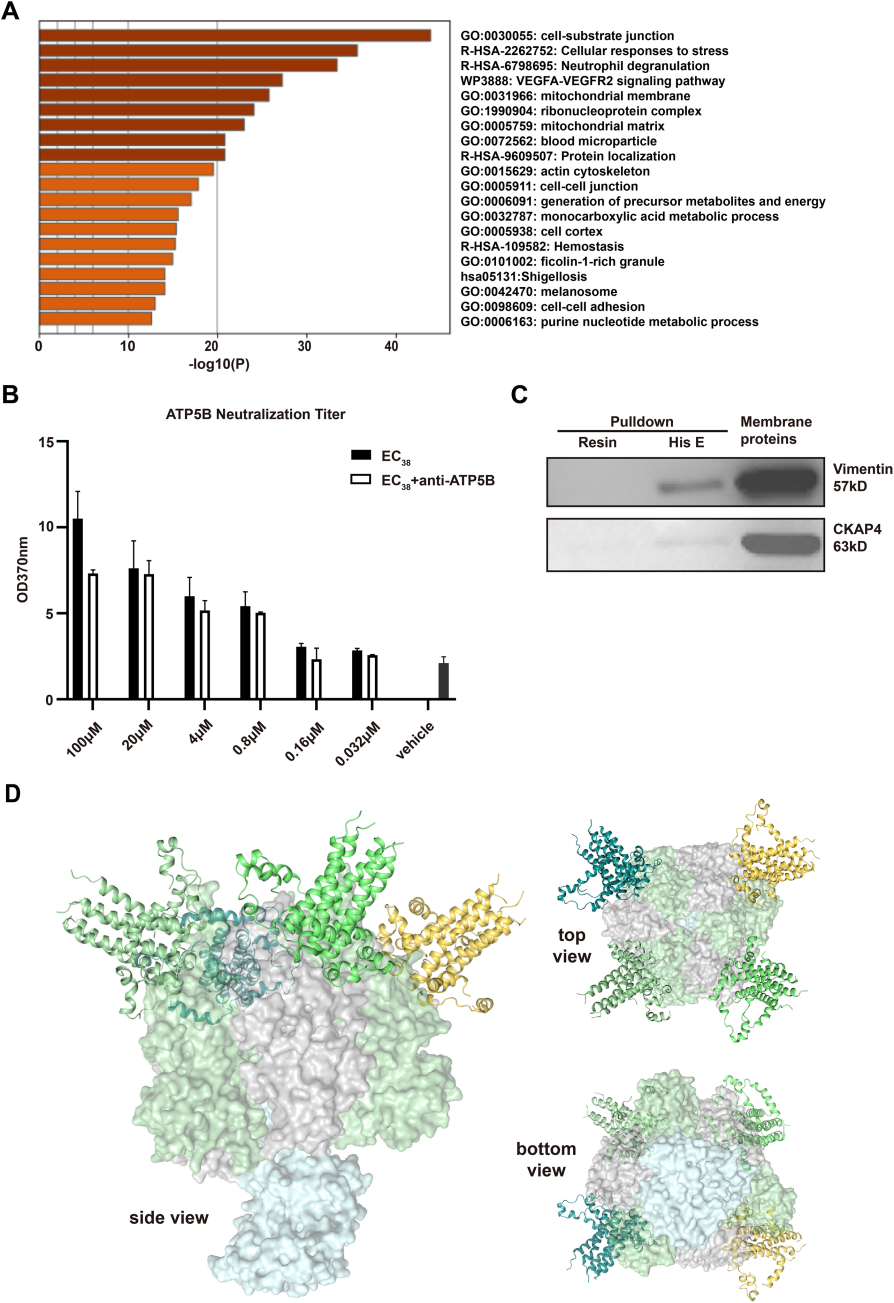
The exterior segment of E-proteins binds a variety of membrane-bound proteins in HUVECs. **(A)** Metascape analysis of the membrane proteins pulled down by EC_38_ peptides. **(B)** ELISA assessment of the blocking ability of anti-ATP5B serum on the binding of EC_38_ peptides onto HUVECs. **(C)** Co-immunoprecipitation in combination with Western blot to verify the interaction of EC_38_ peptides with CKAP4 and vimentin. EC_38_-pulled fractions were resolved in SDS-PAGE and blotted with anti-vimentin or anti-CKAP4 proteins. **(D)** Docking of E-protein viroporins on the F1-ATPase complex. The alpha subunits of ATP synthase are colored with a green surface, while the beta subunits are gray, and the remaining subunits are illustrated with a cyan surface. E-protein pentamers are shown as cartoon models.

To explore the patterns or features of proteins in HUVECs potentially interacting with the EC_38_ segment, EC_38_ peptide-conjugated beads were used to pulldown proteins from HUVEC proteins prepared from all the non-nuclear compartments including various membranes and cytosol. After mass spectrometry assay of the bound protein fractions and data processing, 50 proteins demonstrating the highest −10lgP values (hereafter referred to as top 50) and 112 proteins annotated by Metascape as being or containing components within the “plasma membrane” under “subcellular location” (hereafter referred to as PM112) were listed in **Supplementary Tables 3**, **S4**, respectively. While it is well documented that the membrane system in cells might be tightly conjugated with the cytoskeleton, it was not surprising that some of these proteins were closely related to the cytoskeleton, such as cytoskeleton-associated protein 4 (CKAP4), which is included in the top 50 list but not within the PM112 list. Among the PM112 list, “actin cytoskeleton organization” and “cell–cell interactions” were the most significantly enriched functional protein categories (**Figure 4A**). Comparison of the enriched terms for EC_38_-bound proteins (PM112 plus top 50 sets; **Supplementary Table 3**, **S4**) with those for EC_38_-regulated genes (**Supplementary Table 2**) disclosed 11 shared gene ontology terms, including “epithelial cell differentiation,” “cell–cell adhesion,” “hemostasis,” “viral infection pathways,” “cytokine signaling in immune system,” etc. (**Supplementary Figure 2**), implying that EC_38_ peptides or E-pr might exert biological effects on the endothelium of the human vascular system via these pathways. Without further pursuing these pathways, specific attention was directed toward three non-classical membrane-associated proteins, namely, CKAP4, vimentin, and ATP5B (**Supplementary Table 4**), and their binding interactions with EC_38_ peptides were confirmed by Western blot or ELISA (**Figures 4B, C**). In line with the hypothesis of EC_38_–ATP synthase coupling in HUVECs, random docking of the predicted 3D structure of E-pr pentamers against ATP synthase suggested that the C-terminal sections tend to bind into the “head” part of the ATP synthase (**Figure 4D**). Specifically, the proposed E-pr pentamer tends to be situated within the cavity between the ATP synthase alpha and beta subunits. Additionally, the C-terminal helix and loop structures of E-pr could extend to form more interactions with the polar and charged surfaces of the ATP synthase complex.

## 4 Discussion

Due to the apparent significance of S-proteins in COVID-19 pathogenesis and for the sake of vaccine development, S-proteins have attracted much more attention than other viral proteins, although earlier studies also demonstrated the significant roles of E-pr in the life cycle of the coronavirus (Xu et al., 2020; Orfali et al., 2021; Kuzmin et al., 2022). As outlined in the Introduction, the orientation of E-pr within viral capsids has not been solidified by any data, and contradictions persist among different hypotheses (Surya et al., 2018). While this project was underway, Verdia-Baguena et al. (2021) and Mehregan et al. (2021) independently reported that E-pr facilitated curvature formation in the lipid bilayer membrane, thus favoring the budding of viral particles during virus replication. Kuzmin et al. further proposed that the Ct of E-pr tended to locate at the positive curvature of the membrane (Kuzmin et al., 2022). At the structure–function level, earlier studies with SARS-CoV-1 showed that deletions of the Ct of E-pr decreased viral replication by approximately 100-fold and abrogated inflammatory cytokine production in the hosts, hence causing the least damage to the lungs of the infected animals (Jimenez-Guardeno et al., 2014). Kumar also identified 34 SARS-CoV-2 mutants harboring large Ct segment (>25 aa) deletions in E-pr, which might be deleterious to the virulence of the virus (Kumar et al., 2021). By solidifying the Nt-in/Ct-out orientation of E-pr on the viroid membrane of intact mature SARS-CoV-2 virus, this study provided experimental evidence to support the rationale that E-pr also plays important roles in host cell–virus interactions.

First, the illustration of the Ct-outward orientation of E-pr on viroid membrane confirmed that, when E-pr is considered as a target for vaccine development, only the EC_38_ segment needs be taken into account. This hypothesis gained support from *in silico* predictions, animal studies, and observations from real-world patients (**Figure 2**). As has been widely recognized, the high variation rates in the hotspot S-protein have raised concerns during the development and application of vaccines against the S-protein (Harvey et al., 2021; Andrews et al., 2022). On the contrary, E-pr manifested a lower variation rate (Alam et al., 2020; Susithra Priyadarshni et al., 2021). Troyano-Hernáez et al. reported that, among 105,276 SARS-CoV-2 sequences collected worldwide from 117 countries, the frequency of the most common mutation S68F in E-pr was 0.2% across all SARS-CoV-2 strains, in contrast to T175M (1.00%) in M-pr, R203K and G204R (37.30%) in N-protein, and D614G (81.50%) in S-protein (Troyano-Hernaez et al., 2021). Although the concept of the E-pr-targeted vaccines has been proposed by different groups, our study was the first to confirm the practicability of such vaccines in both animal experiments and human subjects.

Secondly, by identifying a panel of possible interactors of E-pr in endothelial cells, this study highlighted the potential contribution of E-pr-initiated responses to the hemostasis-related vascular disorders accompanying COVID-19, especially those in critical cases with viremia (Wichmann et al., 2020). During the early stage of the COVID-19 pandemic, coagulation abnormalities (e.g., systemic coagulopathies, disseminated intravascular coagulation, or thrombotic microangiopathy; Thachil et al., 2020; Zhang et al., 2020) observed in COVID-19 patients were suspected to be secondary to infection-induced inflammatory or immune disorders like cytokine storms. The direct consequences of SARS-CoV-2 binding to vascular endothelial cells were later proposed, but the S-proteins were still blamed for inducing such reactions (Conway et al., 2022; Lui et al., 2024; Perico et al., 2024). Recently, studies started recognizing E-pr as a potential ligand for some functional cell surface molecules (not including those reported to be in tight junctions; Chai et al., 2021), such as TLR2 on macrophages (Zheng et al., 2021) or CD36 on platelets (Tang et al., 2023). While this manuscript was in preparation, Duan et al. reported that TLR1 on alveolar monocytes/macrophages mediated the pro-inflammatory response to E- and M-pr (Duan et al., 2024). However, none of TLR1, TLR2, or CD36 was pulled down by EC_38_ peptides from HUVECs in the current study. Notably, at least nine proteins (AHNAK, DLG1, ERBIN, EZR, PARD, PDLIM5, SCRIB, SIPA1L3, and TJP2) in the 112 potential interactors contained PDZ domains, which could be readily bound by the DLLV motif at the end of E-pr.

Among all the potential E-pr receptors identified, proteins not previously annotated as “membrane proteins,” such as CKAP4, which demonstrated the highest probability in EC_38_-bound proteins (**Table 2**), warranted extra attention. To the best of our knowledge, CKAP4 has been observed at the cell surface (Bhavanasi et al., 2016) and has been found among proteins in primary human T cells that interacted with HIV-1 gp41 and gp160 proteins. Vimentin is another example that was originally thought to be located inside the cells but was later found to be membrane-bound or even present in the extracellular space (Bucki et al., 2023). Vimentin has been reported to participate in endothelial response to SARS-CoV-2 stimulation in an ACE2-independent manner (Amraei et al., 2022). The third protein that deserved a specific discussion was the ATP synthase or its subunits ATP1F5B or ATP5B (**Table 2**, **Figure 4**). Although the ATP synthase has been traditionally regarded as mitochondria-restricted, recent evidence showed that it is translocated to the outer surface of the cytoplasmic membrane in several types of cells, including cancer cells and endothelial cells, to act as receptors of a variety of physiological molecules (e.g., angiostatin, apolipoprotein A-I, kringle 1–5 of plasminogen, pigment epithelium-derived factor, etc.) (Taurino and Gnoni, 2018). Ectopic ATP synthase has also been shown to bind pathogens like HBV, HIV-1, HEV, or bacterial components like FlimH or flagellar hook (for a detailed reference, see Li et al., 2021). Interestingly, based on the effectiveness of polyphenols in both treating COVID-19 and modulating ATP synthase activity, Panofoli et al. suspected that there might be a link between COVID-19 infection and ectopic ATP synthase (Panfoli and Esposito, 2022). The current study added new evidence supporting the interactions between the SARS-CoV-2 virus and endothelial cells, supposedly including the E-pr-ectopic ATP synthase. However, considering that the distribution of ectopic ATP synthase on plasma membranes and mitochondria was dynamic and correlated with the status of both cells per se and extracellular environments (Panfoli, 2020), the physiological or pathological significance of interactions between E-pr and ectopic ATP synthase in the context of COVID-19 deserves extensive investigation.

**Table 2:** List of the 50 proteins pulled-down by EC_38_ peptides with the highest −10lgP values (top 50).

Gene name	Description	Subcellular location (protein atlas)
CKAP4#	Cytoskeleton-associated protein 4	Unavailable
MYO1C	Unconventional myosin-Ic	Nuclear bodies; plasma membrane (enhanced)
SQOR	Sulfide:quinone oxidoreductase mitochondrial	Mitochondria (supported)
ATP5F1A	ATP synthase subunit alpha mitochondrial	Mitochondria (supported)
MYO1B	Unconventional myosin-Ib	Plasma membrane (enhanced)
LMNA	Prelamin-A/C	Nuclear speckles (supported)
DSP	Desmoplakin	Cell junctions (supported)
HRNR	Hornerin	Mitochondria (uncertain)
HADHA	Trifunctional enzyme subunit alpha mitochondrial	Mitochondria (enhanced)
IQGAP1	Ras GTPase-activating-like protein IQGAP1	Cell junctions; plasma membrane (supported)
HSPA5	Endoplasmic reticulum chaperone BiP	Cytosol (approved)
MYH9	Myosin-9	Actin filaments; plasma membrane (supported); additional: cytosol; nuclear bodies
MYOF	Myoferlin	Vesicles (supported); additional: centriolar satellite; plasma membrane
MSN	Moesin	Plasma membrane (enhanced)
HSPA9	Stress-70 protein mitochondrial	Mitochondria (supported)
SLC25A13	Calcium-binding mitochondrial carrier protein Aralar2	Mitochondria (supported)
HADHB	Trifunctional enzyme subunit beta mitochondrial	Mitochondria (supported)
ITGB4	Integrin beta-4	Cell junctions; plasma membrane (supported)
HSD17B4	Peroxisomal multifunctional enzyme type 2	Peroxisomes (enhanced)
SEPTIN7	Septin-7	Actin filaments; cytosol (supported); additional: midbody; plasma membrane
ENO1	Alpha-enolase	Cytosol; plasma membrane (enhanced)
HSPA8	Heat shock cognate 71 kDa protein	Nucleoplasm (approved); additional: vesicles
UQCRC2	Cytochrome b-c1 complex subunit 2 mitochondrial	Mitochondria (enhanced)
EGFR	Epidermal growth factor receptor	Cell junctions; plasma membrane (supported); Additional: nucleoli; nucleoplasm
ATP5F1B	ATP synthase subunit beta mitochondrial	Mitochondria (enhanced)
RBMX	RNA-binding motif protein X chromosome	Nucleoplasm (supported)
RPN1	Dolichyl-diphosphooligosaccharide--protein glycosyltransferase subunit 1	Endoplasmic reticulum (enhanced); additional: cytosol
SEPTIN9	Septin-9	Actin filaments (enhanced)
ATAD3B	ATPase family AAA domain-containing protein 3B	Mitochondria (enhanced)
VIM	Vimentin	Intermediate filaments (supported)
AHNAK	Neuroblast differentiation-associated protein AHNAK	Cytosol; plasma membrane (enhanced)
TUFM	Elongation factor Tu mitochondrial	Mitochondria (enhanced)
TXNDC5	Thioredoxin domain-containing protein 5	Endoplasmic reticulum (approved)
LTF	Lactotransferrin (fragment)	
UQCRC1	Cytochrome b-c1 complex subunit 1 mitochondrial	Mitochondria (supported)
SF3A1	Splicing factor 3A subunit 1	Nuclear speckles; nucleoplasm (enhanced)
MYO1E	Unconventional myosin-Ie	Nucleoplasm; plasma membrane (approved); additional: cytosol
AGK	Acylglycerol kinase mitochondrial	Mitochondria (supported); additional: vesicles
RBMXL1	RNA-binding motif protein X-linked-like-1	Nucleoplasm (approved)
HK1	Hexokinase-1	Mitochondria (supported)
ACAA2	3-ketoacyl-CoA thiolase mitochondrial	Mitochondria (supported)
PKP1	Plakophilin-1	Nucleoplasm; plasma membrane (approved)
JUP	Junction plakoglobin	Cell junctions; plasma membrane (supported); additional: vesicles
IQGAP3	Ras GTPase-activating-like protein IQGAP3	Nucleoli rim (approved); additional: mitotic chromosome; nucleoplasm
LMNB1	Lamin-B1	Nuclear membrane (supported)
HNRNPU	Heterogeneous nuclear ribonucleoprotein U	Nucleoplasm (supported)
SDHA	Succinate dehydrogenase [ubiquinone] flavoprotein subunit mitochondrial	Mitochondria (supported); additional: nucleoli
DDOST	Dolichyl-diphosphooligosaccharide--protein glycosyltransferase 48 kDa subunit	Endoplasmic reticulum (supported)
HSPA1A	Heat shock 70 kDa protein 1A	Nucleoplasm; vesicles (approved); additional: cytosol
GLUD1	Glutamate dehydrogenase 1 mitochondrial	Mitochondria (supported)

More information about the genes is given in **Supplementary Table 4**. #Genes in green denoted the proteins identified as E-pr interactors in the reference—Samavarchi-Tehrani et al. (2020). Genes in red highlighted the proteins denoted to be (partially) distributed in the plasma membrane.

Finally, because blood pressure elevation is being recognized as a significant immediate and delayed consequence of natural COVID-19 infection or vaccination (Angeli et al., 2022; Bielecka et al., 2024), it is proper to highlight the findings that “GO:0001990, regulation of systemic arterial blood pressure by hormone” was significantly enriched in EC_38_-regulated genes (**Table 1**). Due to the innate link between the S-protein and ACE2, earlier investigations into COVID-19-associated hypertension largely focused on this pathway (Sardu et al., 2020; Wang et al., 2021; Gholami et al., 2023). Current findings added E-pr as a new suspect for COVID-19-induced hypertension.

In summary, this study used experiments to demonstrate that E-pr adopts a Ct-out orientation on the surface of the SARS-CoV-2 virus, allowing the 38-aa-long exterior segment to participate in virus–host interactions in other ways than previously known (i.e., in ERGIC inside the cells). E-pr might mediate the adhesion of exogenous virus to cellular surface molecules (like CKAP4 and ATP5B) or facilitate newly generated E-pr or virus inside the cells to bind cytosol-residing partners to modulate the functions of host cells (as reflected by the transcriptome changes). Recent studies suggested that the number of spikes on the viroid surface (approximately 24 ± 9 per virion; Ke et al., 2020) might be much lower than previously thought (approximately 100 copies; Bar-On et al., 2020). Hence, the sparsity of the large spikes (approximately 20 nm from the virus capsid planar surface) over the virion surface provided the short EC_38_ segments (estimated at 5.3 nm) more stereo spaces to interact with their hypothetical interactors on the host cell surface (**Supplementary Figure 3**). However, extensive investigations are warranted to confirm the significance of these hypothetical interactions in the context of actual infection, in either the vascular endothelium or other cells or tissues. Answers to these questions might lead to strategies utilizing E-pr as a target to block exogenous virus from attaching to host cells or to interfere with stimulus-initiated signaling in host cells. Collectively, dissection of the whole pathway, from the envelope protein in SARS-CoV-2 to host receptors for E-pr and to responding genes, might help find new solutions to the COVID-19 pandemic.

## Data Availability

The data supporting the findings of this study are available from the authors upon request without reservation. The RNA-seq data generated in this study have been deposited in the NCBI Gene Expression Omnibus (GEO) database under the accession code GSE268369 and can be accessed at: https://www.ncbi.nlm.nih.gov/geo/query/acc.cgi?acc=GSE268369.
