# Supplementary material for: C-terminus-outward orientation of SARS-CoV-2 envelope proteins on viral capsid enables a novel virus–cell interaction pathway
Source: Front Cell Infect Microbiol. 2026 Mar 12;16:1776252. doi: 10.3389/fcimb.2026.1776252 (PMC13018128; doi:10.3389/fcimb.2026.1776252)
Supplement: Supplementary file 1 [file DataSheet1.pdf]

Supplementary Information

C-terminus-outward orientation of SARS-CoV-2 envelope proteins on viral capsid enables a novel virus-cell interaction pathway

Jie Xu[1], Wei Zhao[1], Yuanyuan Li[2], Jing Zheng[3], Yulian Wang[1], Huimin Sun[4], Sijin Wu[5], Baoqing Fu[4], Yiqiang Wang[1,4,5,*]

[1] Eye Institute of Xiamen University, School of Medicine, Xiamen University, Xiamen, China.
[2] Department of Laboratory Examination, People's Hospital of Rizhao City, Rizhao, China.
[3] Xiamen Center for Diseases Control and Prevention, Xiamen, China.
[4]Mecial Center and Xiang'an Hospital of Xiamen University, Xiamen University, Xiamen, China.
[5] Wisdom Lake Academy of Pharmacy, Xi'an Jiaotong-Liverpool University, Suzhou, China.

***Corresponding author:** Yiqiang Wang, Wisdom Lake Academy of Pharmacy, Xi'an Jiaotong-Liverpool University, 111 Ren’ai Road, Suzhou 215123, China. Tel: 86-512-88970213; Email: Yiqiang.Wang@xjtlu.edu.cn. ORCID: 0000-0003-1552-7529

SUPPLEMENTARY MATERIALS AND METHODS

Protein or synthetic peptides

Recombinant SARS-CoV-2 S-trimer proteins（with 6xHis at the C-terminal）were from the commercial resource (Cat# DRA49, MW136.6 kDa; Novoprotein Company, Suzhou, China). E-protein associated peptides and M peptide, either tagged a at C-terminal with 6xHis or conjugated with FITC at the N-terminus, were ordered from Bootech Bioscience and Technology (Shanghai, China. All peptides were dissolved in sterile PBS. Their sequences were as follows:

Peptides used for immunization:

MN_{19}:	MADSNGTITVEELKKLLEQ
EC_{38}:	RLCAYCCNIVNVSLVKPSFYVYSRVKNLNSSRVPDLLV

Peptides for measurement of murine antibodies:

MN_{19}-His:	MADSNGTITVEELKKLLEQ-HHHHHH:
EC_{38}-His:	RLCAYCCNIVNVSLVKPSFYVYSRVKNLNSSRVPDLLV-HHHHHH

Peptides used for binding assay onto HUVEC cells:

EO:	FITC-RLCAYCCNIVNVSLVKPSFYVYSRVKNLNSSRVPDLLV
EN:	FITC-RLCAYCCNIVNVSLVKPSF
EM:	FITC-VSLVKPSFYVYSRVKN
EC:	FITC-YVYSRVKNLNSSRVPDLLV
EL:	FITC-RLCAYCCNIVNVSLVKPSFYVYSRVKNLNSSRVP

Animal usage and housing

All experimental procedures involving animals were reviewed and approved (File# XMULAC20201004) by the Animal Welfare and Ethic Committee of Xiamen University following the Guidelines on the Humane Treatment of Laboratory Animals (Ministry of Science and Technology of China, 2006). Mice were purchased from the SLAC Animal Center (Shanghai, China) and housed in the specific pathogen-free (SPF) facility of Animal Center of Xiamen University (Xiamen, China) on a 12/12-hour light/dark cycle with standard laboratory rodent chow and drinking water ad libitum.

Generation of murine anti-EC_{38} antiserum

C57BL/6 mice (n = 4/group, male) were used for this study. An immunization protocol that had been proven to work well where 10 µg (i.e. 18.18 pmol) spike protein trimers (S-trimer) induced significant immune response (not discussed in current report) were modified to fit in current project. In brief, mice were immunized with 200 µL mixture containing 0.28 µg (i.e. 54.54 pmol, namely three folds of molar dose corresponding to 10 µg S-trimer) or 1.4 µg (i.e. 272.7 pmol, five folds of low dosing) of EC_{38}, 20 µg CpG-ODN 1018 (sequence 5′-TGACTGTGAACGTTCGAGATGA-3′, with phosphorothioate backbone) plus 100 µL of Incomplete Freund Adjuvant (IFA, Beyotime, Shanghai, China). For comparison, other mice were immunized with same doses of MN_{19} peptides (i.e. 54.54 pmol = 0.16 µg, or 272.7 pmol = 0.80 µg) in identical adjuvant mixtures. The dosing of 54.54 pmol for EC_{38} and MN_{19} were adapted from In all mice, injections were distributed in four footpads (20 µL/foot) plus two subcutaneous injection (60 µL/site) at each side of the back. After 3 weeks, boost immunization was achieved with half dosing antigen plus IFA at all six sites. After 3 more weeks, boost dosing was repeated but at four flank sites only. Five weeks after last dosing, animals were anesthesia with CO_2, and blood were collected from retro-orbital space for anti-sera preparation. Then, mice remaining in anesthesia were euthanatized by neck dislocation.

ELISA measurement of antibody titers against EC_{38}, MN_{19} in sera of immunized mice

Titers of hypothetical antibodies in sera from above immunized mice were measured with ELISA. In brief, 96-well plates (Corning, Corning, NY) were coated with rabbit Anti-6X His tag® antibody (ab9108, Abcam) at 1 µg/mL, 50 µL/well, room temperature overnight and blocked with 5% non-fat milk at room temperature overnight. After blocking, plates washed with PBST (PBS containing 0.1% Tween-20) and then incubated

with EC_{38}-His or MN_{19}-His peptides (0.07 μM, 50 μL/well). After incubating for 1h at room temperature, the plates were washed four times with PBST. Then serial dilutions of murine antisera (1:50) were added to the wells (50 μL/well) for 1h at room temperature, and the plates were washed four times with PBST, followed by incubating with 50 μL/well species specific secondary antibodies (all from Abcam), e.g. 1:5000 diluted HRP conjugated goat anti-mouse IgM (ab98679, Abcam) or IgG2a (ab98698, Abcam), IgG2b (ab98703, Abcam) antibodies at RT for 60 min. Plates were washed four times with PBST and signals were developed using TMB substrate (Beyotime) for 1 h. The optical density (OD) was measured directly at 370 nm using a 1510 Multiskan Go Spectrophotometer (Thermo Fisher Scientific, MA).

ELISA measurement of antibody titers against EC_{38}, MN_{19}, or S-trimers in COVID-19 patients or healthy controls

Sera for antibody detection were prepared as described in text. ELISA plates for mearing the antibodies were prepared as detailed in above section. The plates were coated with S-trimer (0.002 μM, 50 μL/well) or the EC_{38}, MN_{19} peptides (0.07 μM, 50 μL/well) respectively for 1h at room temperature, followed by 4 washes with PBST. After incubation of human sera dilutes in coated plates and washing with PBST, 1:10,000 diluted goat anti-human IgM-HRP(ab98549, Abcam), 1:1,000 diluted mouse anti-human IgG1-HRP(ab99774, Abcam), 1:1,000 diluted mouse anti-human IgG2-HRP(ab99779, Abcam) were used for incubation at RT for 60 min. Plates were then washed three times with PBST and signals were developed using TMB substrate (Beyotime) for 30 min. Stop Solution for TMB Substrate (Beyotime) was applied, and the optical density of the products was read at 630 nm.

Cell lines and culture

HEK293T-ACE2hR cells were cultured at 37°C and 5% CO2 in Dulbecco's modified Eagle's medium (Gibco, Grand Island, NY) supplemented with 10% heat-inactivated fetal bovine serum (ABW, Vancouver, Canada). The medium for the 293T-hACE2 cells additionally contained 5μg/ml blasticidin (Basal Media, Shanghai, China), 100 IU/mL penicillin and 100 μg/mL streptomycin (Biological Industries, BEIT HAEMEK, Israel). Human umbilical vein endothelial cells (HUVEC) were cultured at 37°C and 5% CO2 in Dulbecco's modified Eagle's medium (Gibco) supplemented with 10% heat-inactivated fetal bovine serum (ABW). All mediums were supplemented with 100 IU/mL penicillin and 100 μg/mL streptomycin (Biological Industries).

Confirmation of binding of EC_{38} peptides with CKAP4 and vimentin

100 μM His-tagged synthetic E protein (400 μL) was added to 100 μL 50% HIS-tag Purification Resin (Beyotime), incubated at 4℃ for 60 min, washed twice with 200 μL wash buffer (50mM NaH_2PO_4, 300 mM NaCl and 2 mM imidazole, pH 8.0). Then added 100 μg HUVEC cells membrane protein and incubated at 4 ℃ for 120 min. After three washes with 200 μL wash buffer, proteins were released with elution buffer (50 mM NaH_2PO_4, 300 mM NaCl and 50 mM imidazole, pH 8.0). The eluted proteins were separated by 10% SDS-PAGE in Tris-HCl buffer and then transferred onto PVDF membranes. After blocking for 1h at room temperature in 10% (w/v) skimmed milk, membranes were incubated with antibodies against vimentin (sc-373717, Santa Cruz Biotechnology,1:500), CKAP4 (sc-393544, Santa Cruz Biotechnology, 1:500) over night at 4℃. Then the membranes were washed three times with TBST (20 mM Tris-HCl, 150 mM NaCl, 0.1% Tween 20, pH 7.5). Next, membranes were incubated with HRP-linked rabbit anti-mouse secondary antibody (ab6728, Abcam, 1:5000) for 1h at room temperature. Signals were detected with BeyoECL Plus (Beyotime) and recorded by the transilluminator (ChemiDoc XRS System; Bio-Rad, Philadelphia, PA).

SUPPLEMENTARY RESULTS

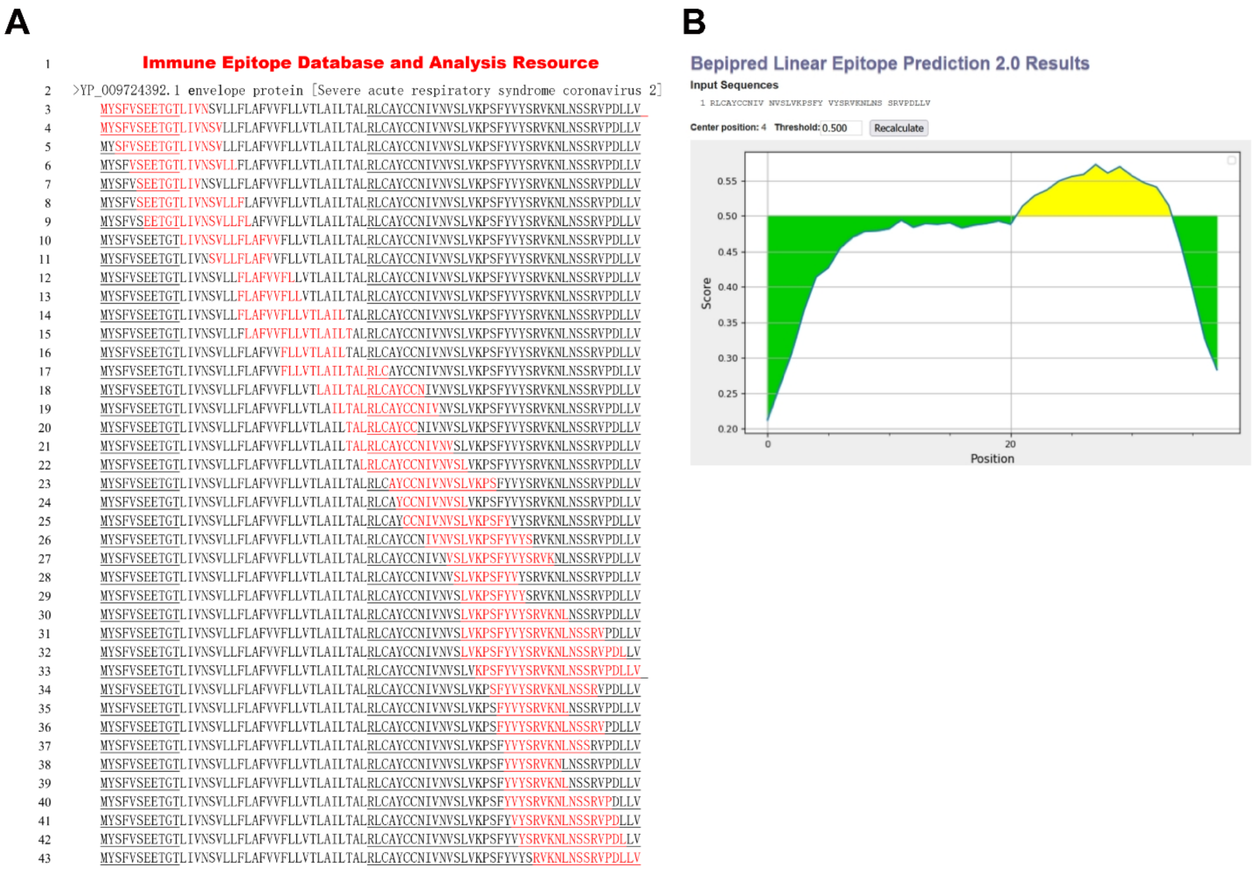

Figure S1. Division and immunogenicity of the E-protein of SARS-CoV-2 virus. Prediction of hypothetical B-epitopes by Immune Epitope DataBase and Analysis Resource (https://www.iedb.org) revealed 41 potential and overlapping B-epitopes (red font, A) and the summative antigenicity score (B) across the whole length of E-protein. Underlined sections represented the free terminals while the un-underlined segment the transmembrane domain.

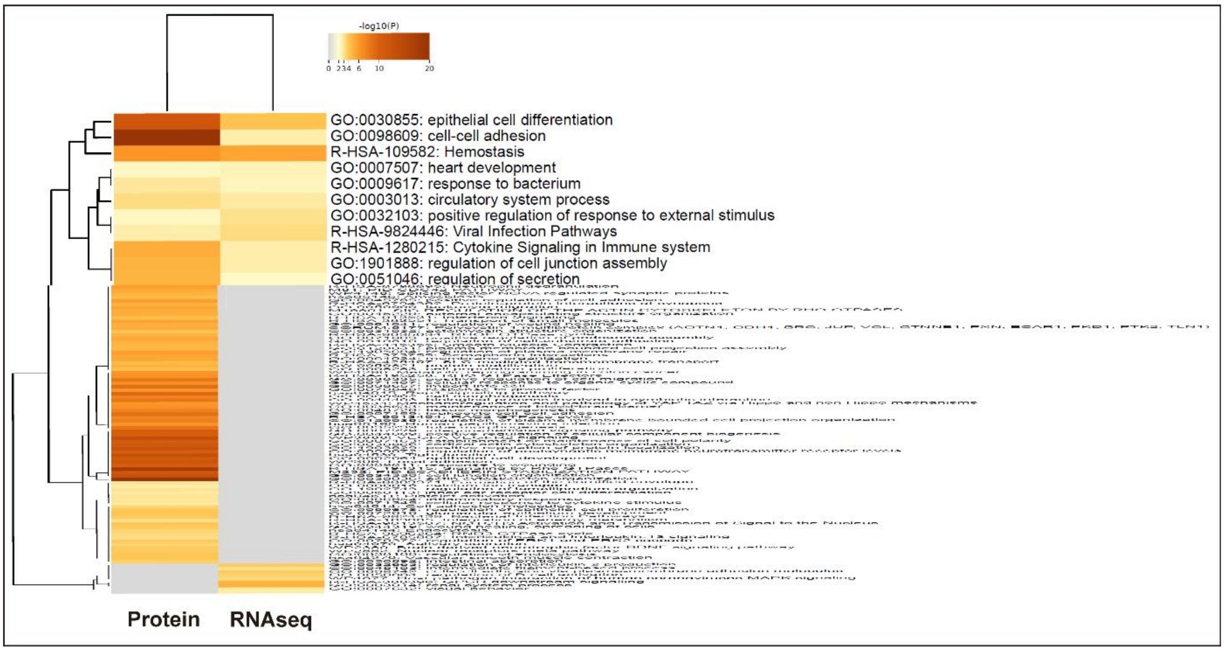

Figure S2. Clustering of the genes in EC_{38}-regulated transcriptome and EC_{38}-peptide-bound membrane proteins. Squeezed sections in lower part represented those GO terms enriched in single one sample.

Table S1. List of DEGs manifesting up- or down regulation in HUVEC upon treatment with EC_{38} peptides.

Gene Symbol	EC_{38} (E-protein)	Control	EC_{38}/Control
UP-regulated			
GAGE12G	0.517±0.021	0±0	~ ∞
PNMT	0.173±0.075	0±0	~ ∞
GLT8D2	0.13±0.026	0±0	~ ∞
PCDHGA5	0.073±0.015	0±0	~ ∞
CEACAM8	0.053±0.023	0±0	~ ∞
TMEM45B	0.05±0.01	0±0	~ ∞
DOCK8	0.043±0.023	0±0	~ ∞
B3GALT2	0.04±0.017	0±0	~ ∞
ACTN2	0.027±0.012	0±0	~ ∞
ADAMTS18	0.027±0.012	0±0	~ ∞
CORIN	0.02±0	0±0	~ ∞
DNAH11	0.01±0	0±0	~ ∞
H2BC14	1.64±0.888	0.143±0.248	11.44
SVOPL	0.137±0.032	0.013±0.012	10.25
POTED	0.033±0.006	0.003±0.006	10.00
RASL10A	0.33±0.104	0.033±0.029	9.90
MAP2K6	0.03±0	0.003±0.006	9.00
PLCL1	0.033±0.012	0.007±0.012	5.00
ZNF418	0.12±0.056	0.027±0.006	4.50
MEP1B	0.057±0.012	0.013±0.023	4.25
CHRNB2	0.073±0.006	0.02±0.01	3.67
C4orf36	0.647±0.012	0.177±0.098	3.66
IFITM1	0.527±0.142	0.15±0.13	3.51
CGB7	0.643±0.075	0.207±0.064	3.11
POU4F3	0.08±0	0.027±0.023	3.00
SPATA25	0.753±0.175	0.253±0.095	2.97
GNAT2	0.203±0.038	0.07±0.07	2.90
NAPSA	0.277±0.092	0.107±0.046	2.59
GPR83	0.083±0.012	0.033±0.029	2.50
MYO1H	0.183±0.012	0.077±0.006	2.39
SHC2	0.257±0.078	0.11±0.02	2.33
NBPF7	0.69±0.07	0.33±0.195	2.09
FOXD4L1	0.347±0.055	0.167±0.059	2.08
LOC102724200	1.95±0.13	0.943±0.475	2.07
PRR4	5.823±1.397	2.89±0.607	2.01
ANKLE1	0.187±0.021	0.093±0.021	2.00
MPL	0.107±0.006	0.053±0.025	2.00
EPPK1	0.027±0.006	0.013±0.006	2.00
AMT	0.823±0.215	0.42±0.03	1.96
SUCNR1	0.137±0.006	0.07±0.02	1.95
PLB1	0.077±0.015	0.04±0	1.92
SBK3	0.593±0.047	0.313±0.055	1.89
CYP39A1	0.157±0.029	0.083±0.029	1.88
KRT17	1.503±0.196	0.807±0.168	1.86
LOC105370579	0.5±0.072	0.27±0.095	1.85

CD34	0.067±0.006	0.037±0.012	1.82
LOC105369669	0.703±0.067	0.39±0.125	1.80
COL9A3	0.18±0.017	0.1±0.035	1.80
FAM20C	0.567±0.072	0.32±0.075	1.77
FLRT3	0.2±0.035	0.113±0.032	1.76
FAM71F2	0.3±0.046	0.173±0.046	1.73
HEY1	0.427±0.076	0.247±0.076	1.73
SERPINF2	0.23±0.052	0.133±0.029	1.73
CASP1	1.277±0.081	0.75±0.217	1.70
FXYD6	0.527±0.029	0.313±0.06	1.68
C2orf88	1.187±0.145	0.723±0.04	1.64
XKR9	3.327±0.065	2.04±0.442	1.63
PTGIR	0.43±0.072	0.267±0.059	1.61
TMEM150C	0.89±0.105	0.557±0.14	1.60
P2RY1	0.063±0.006	0.04±0.01	1.58
LOC112268350	0.613±0.074	0.39±0.085	1.57
NLRC5	0.33±0.053	0.21±0.04	1.57
SLC16A6	0.213±0.031	0.137±0.025	1.56
CAPS2	0.597±0.038	0.383±0.055	1.56
PAQR5	0.197±0.032	0.127±0.029	1.55
BHLHB9	0.88±0.105	0.57±0.111	1.54
CCDC73	0.323±0.006	0.21±0.036	1.54
KIF21B	0.21±0.01	0.137±0.032	1.54
RUNX2	0.21±0.01	0.137±0.042	1.54
DNASE1L2	1.14±0.12	0.75±0.14	1.52
COBL	0.247±0.047	0.163±0.021	1.51
FLVCR2	0.6±0.061	0.4±0.101	1.50
MPZL2	0.14±0.017	0.093±0.023	1.50
UST	0.94±0.044	0.627±0.172	1.50
DOWN-regulated			
APOBEC3F	5.457±0.732	8.23±0.701	0.66
LOC102724642	0.707±0.13	1.067±0.116	0.66
SDCBP2	1.397±0.287	2.157±0.121	0.65
ID2	0.987±0.136	1.543±0.286	0.64
NPFF	2.973±1.025	4.7±0.137	0.63
RAB19	1.29±0.173	2.04±0.239	0.63
H3C8	12.137±0.575	19.403±1.517	0.63
TTC25	0.2±0.035	0.323±0.057	0.62
NLGN1	0.07±0.01	0.113±0.023	0.62
SYNC	0.28±0.082	0.463±0.067	0.60
PDE8B	0.227±0.025	0.407±0.032	0.56
RIPOR3	0.673±0.047	1.227±0.267	0.55
H2AC13	3.063±0.28	5.653±1.46	0.54
EFNA2	0.127±0.035	0.247±0.05	0.51
VN1R1	0.137±0.065	0.273±0.006	0.50
SPAG8	0.063±0.006	0.127±0.038	0.50
TMEM140	1.14±0.123	2.307±0.343	0.49
ANKRD34B	0.11±0.017	0.223±0.051	0.49
PCSK5	0.173±0.038	0.37±0.035	0.47

NOD2	0.09±0.036	0.193±0.032	0.47
OPRL1	0.313±0.05	0.697±0.11	0.45
PDE4A	0.123±0.021	0.28±0.026	0.44
H3C2	0.743±0.371	1.697±0.445	0.44
FAM209B	0.467±0.09	1.097±0.214	0.43
ZNF154	0.08±0.01	0.19±0.046	0.42
APOM	0.73±0.308	1.817±0.242	0.40
TXNDC2	0.06±0.044	0.15±0.017	0.40
H3C1	1.257±0.45	3.307±0.408	0.38
ABI3BP	0.013±0.012	0.037±0.006	0.36
PSG7	0.103±0.045	0.327±0.121	0.32
GIMAP2	0.05±0.044	0.173±0.04	0.29
CATSPER1	0.09±0.044	0.32±0.062	0.28
CCER2	0.067±0.065	0.26±0.07	0.26
CAMKV	0.053±0.04	0.21±0.035	0.25
MRO	0.013±0.012	0.053±0.012	0.25
SMIM10	0.073±0.075	0.293±0.075	0.25
KRT34	0.04±0.035	0.183±0.06	0.22
CCR4	0.033±0.058	0.163±0.006	0.20
RNF148	0.03±0.052	0.15±0.052	0.20
CALML6	0.06±0.026	0.317±0.15	0.19
CD72	0.027±0.046	0.147±0.012	0.18
SIGLEC16	0.017±0.029	0.1±0.03	0.17
SYCE1L	0.09±0.078	0.57±0.269	0.16
SYNE4	0.027±0.046	0.217±0.098	0.12
IFITM2	0.063±0.11	0.57±0.18	0.11
KLHL6	0.003±0.006	0.03±0	0.11
TMEM236	0.007±0.012	0.083±0.006	0.08
FAM47E	0±0	0.067±0.006	0.00
RDH12	0±0	0.053±0.006	0.00
CEACAM5	0±0	0.033±0.006	0.00
SORCS2	0±0	0.023±0.006	0.00
RAB17	0±0	0.083±0.023	0.00
BTK	0±0	0.053±0.015	0.00
TMEM239	0±0	0.11±0.035	0.00
ARHGEF37	0±0	0.033±0.012	0.00
CR1	0±0	0.043±0.015	0.00
SPEGNB	0±0	0.217±0.09	0.00
PCDHB4	0±0	0.04±0.017	0.00
RNASE7	0±0	0.107±0.046	0.00
TMC1	0±0	0.027±0.012	0.00
IL1A	0±0	0.067±0.029	0.00
ZNF385C	0±0	0.043±0.023	0.00
ST6GALNAC2	0±0	0.127±0.076	0.00
ANO1	0±0	0.02±0	0.00
LOC107985022	0±0	0.13±0	0.00
RASGEF1A	0±0	0.03±0	0.00

Table S2. List of genes manifesting up- or down regulation in HUVEC upon treatment with MN_{19} peptides.

Gene Symbol	MN_{19} (M-protein)	Control	MN_{19}/Control
UP-regulated			
H2AC12	1.143±0.275	0±0	~ ∞
LPA	0.017±0.006	0±0	~ ∞
B3GALT2	0.05±0.017	0±0	~ ∞
ARMC12	0.14±0.052	0±0	~ ∞
ARGFX	0.027±0.012	0±0	~ ∞
DOCK8	0.033±0.015	0±0	~ ∞
ACTA1	0.08±0	0±0	~ ∞
ANGPTL2	0.03±0	0±0	~ ∞
CACNA1D	0.01±0	0±0	~ ∞
LOC101928268	0.13±0	0±0	~ ∞
SULT1C4	0.04±0	0±0	~ ∞
TLR9	0.043±0.012	0.007±0.012	6.50
HPX	0.117±0.04	0.023±0.04	5.00
KCNV2	0.083±0.029	0.017±0.029	5.00
ZCCHC12	0.083±0.029	0.017±0.029	5.00
MAP4K1	0.067±0.023	0.013±0.023	5.00
FTCD	0.05±0.017	0.01±0.017	5.00
SUSD3	0.2±0.07	0.04±0.069	5.00
DDX43	0.13±0.04	0.027±0.023	4.88
CRLF2	0.113±0.029	0.023±0.04	4.86
SMCO2	0.123±0.006	0.027±0.025	4.63
CYRIA	0.117±0.031	0.027±0.031	4.38
PILRA	0.123±0.006	0.03±0.052	4.11
H3C15	4.83±0.555	1.197±2.073	4.04
LOC105376714	0.653±0.125	0.167±0.194	3.92
NPY4R2	0.05±0.01	0.013±0.012	3.75
C4orf36	0.62±0.125	0.177±0.098	3.51
GNA15	0.2±0.026	0.06±0.052	3.33
APOL3	0.16±0.05	0.05±0	3.20
CLEC4A	0.24±0.035	0.08±0.036	3.00
UNC5C	0.02±0	0.007±0.006	3.00
POLN	0.277±0.051	0.093±0.065	2.96
NBPF7	0.967±0.254	0.33±0.195	2.93
CFAP97D1	0.087±0.021	0.03±0	2.89
GRIK1	0.057±0.012	0.02±0.017	2.83
CSF2RA	1.113±0.332	0.403±0.102	2.76
TP53TG5	0.61±0.04	0.223±0.15	2.73
KRT32	0.55±0.061	0.207±0.144	2.66
MRGPRX3	0.157±0.038	0.063±0.006	2.47
EIF4E3	0.13±0.017	0.053±0.029	2.44
UCP3	0.417±0.1	0.177±0.045	2.36
GJA9	0.14±0	0.06±0.026	2.33
ALDH8A1	0.22±0.04	0.1±0.026	2.20
OVGP1	0.433±0.045	0.2±0.052	2.17
ASB9	0.81±0.087	0.377±0.127	2.15
WNT16	0.12±0.017	0.057±0.023	2.12

LYPD1	0.43±0.095	0.203±0.07	2.11
TCTEX1D4	0.743±0.172	0.353±0.159	2.10
C1orf229	0.337±0.08	0.167±0.068	2.02
TSNAXIP1	0.577±0.061	0.287±0.096	2.01
LRRC19	0.073±0.012	0.037±0.012	2.00
HEY1	0.493±0.064	0.247±0.076	2.00
LOC107985524	0.187±0.012	0.093±0.045	2.00
GDF7	0.027±0.006	0.013±0.006	2.00
ADAM32	0.25±0.035	0.127±0.006	1.97
MEIOC	0.22±0.044	0.113±0.023	1.94
LONRF2	0.183±0.04	0.097±0.015	1.90
TUBAL3	0.2±0	0.107±0.04	1.88
ZNF483	1.863±0.309	0.997±0.047	1.87
LHX2	0.277±0.067	0.15±0.026	1.84
H4C5	4.02±0.803	2.187±0.713	1.84
CRPPA	0.183±0.012	0.1±0.03	1.83
MXRA8	0.777±0.117	0.437±0.025	1.78
CCDC7	0.463±0.075	0.263±0.075	1.76
B3GALT5	0.527±0.067	0.3±0.061	1.76
SPON1	0.25±0.053	0.143±0.031	1.74
CBLN3	0.447±0.023	0.257±0.087	1.74
PDGFRL	0.62±0.044	0.357±0.14	1.74
INHA	0.37±0.052	0.217±0.04	1.71
KIAA1614	0.33±0.07	0.193±0.029	1.71
ZNF596	0.687±0.035	0.41±0.155	1.67
ARSG	1.02±0.192	0.613±0.012	1.66
ZCCHC18	0.51±0.046	0.307±0.049	1.66
LOC112268350	0.647±0.108	0.39±0.085	1.66
LBX1	1.097±0.091	0.67±0.141	1.64
NLRC5	0.34±0.046	0.21±0.04	1.62
LOC112268092	0.333±0.006	0.21±0.05	1.59
GRIN1	0.28±0.017	0.177±0.04	1.58
FAM167A	0.3±0.05	0.19±0.035	1.58
SHROOM3	0.273±0.046	0.173±0.032	1.58
LRCH2	0.383±0.075	0.243±0.025	1.58
MYO1H	0.12±0.017	0.077±0.006	1.57
XKR9	3.15±0.221	2.04±0.442	1.54
COBL	0.247±0.015	0.163±0.021	1.51
CTSS	0.523±0.064	0.347±0.072	1.51
CCBE1	0.86±0.087	0.57±0.121	1.51
DOWN-regulated			
SYNDIG1L	0.073±0.012	0.113±0.021	0.65
SLC16A9	0.287±0.076	0.443±0.057	0.65
FMC1	5.497±0.975	8.527±1.114	0.64
PDE4A	0.18±0.053	0.28±0.026	0.64
SIRT4	1.133±0.072	1.777±0.146	0.64
LOC100996747	20.997±3.051	33.02±5.796	0.64
FBXO16	1.587±0.159	2.547±0.391	0.62
MFSD2B	1.163±0.179	1.907±0.301	0.61

SEPTIN1	0.883±0.15	1.45±0.243	0.61
APOBEC3F	4.913±1.835	8.23±0.701	0.60
CYTH4	0.287±0.023	0.483±0.071	0.59
IL4I1	0.217±0.032	0.367±0.059	0.59
LRRIQ3	0.617±0.105	1.063±0.18	0.58
CCR4	0.09±0.017	0.163±0.006	0.55
TTC25	0.177±0.021	0.323±0.057	0.55
ZNF853	0.123±0.04	0.24±0.056	0.51
H3C1	1.687±0.63	3.307±0.408	0.51
RASGRP2	0.31±0.089	0.62±0.13	0.50
LOC391322	0.81±0.245	1.63±0.338	0.50
EFNA2	0.117±0.023	0.247±0.05	0.47
LAIR1	0.183±0.075	0.407±0.012	0.45
TCHH	0.073±0.012	0.163±0.015	0.45
TIGD4	0.08±0.017	0.187±0.047	0.43
ERMN	0.03±0	0.07±0.017	0.43
ACRV1	0.083±0.04	0.217±0.025	0.38
CAMKV	0.08±0.072	0.21±0.035	0.38
CHST5	0.03±0	0.08±0	0.38
SAMD14	0.077±0.015	0.213±0.078	0.36
WNT4	0.083±0.035	0.237±0.065	0.35
FXYD6	0.11±0.101	0.313±0.06	0.35
FAM209A	0.153±0.133	0.45±0.01	0.34
SPDYA	0.07±0.07	0.21±0.017	0.33
C1R	0.18±0.132	0.543±0.081	0.33
ACTN3	0.04±0	0.123±0.05	0.32
DEPP1	0.067±0.076	0.24±0.04	0.28
FXYD7	0.14±0.121	0.533±0.107	0.26
GPNMB	0.04±0.04	0.173±0.06	0.23
EXOC3L4	0.013±0.023	0.063±0.021	0.21
LRRC24	0.06±0.104	0.337±0.081	0.18
RENBP	0±0	0.083±0.006	0.00
FAM47E	0±0	0.067±0.006	0.00
PCDHGB6	0±0	0.067±0.006	0.00
LDB3	0±0	0.07±0.01	0.00
CEACAM5	0±0	0.033±0.006	0.00
SORCS2	0±0	0.023±0.006	0.00
BTK	0±0	0.053±0.015	0.00
SPEGNB	0±0	0.217±0.09	0.00
CD101	0±0	0.04±0.017	0.00
SSX2B	0±0	0.12±0.052	0.00
HOTS	0±0	0.043±0.021	0.00
CLVS1	0±0	0.08±0.046	0.00
NUTM1	0±0	0.03±0.017	0.00
KLHL6	0±0	0.03±0	0.00
TEX45	0±0	0.06±0	0.00
FPGT-TNNI3K	0±0	0.04±0	0.00

Note: Genes in red font denoted those genes that were also seen in EC_{38} treatment (Table S1).

Table S3. List of the 50 proteins pulled-down by EC_{38} peptides with highest -10lgP values (Top50).

Accession	-10lgP	Coverage (%)	#Peptides	#Unique	Gene Name	Description	Subcellular Location (Protein Atlas)
Q07065|CKAP4_HUMAN	456.14	76	59	57	**CKAP4*#**	Cytoskeleton-associated protein 4	unavailable
O00159|MYO1C_HUMAN	445.48	61	99	94	MYO1C	Unconventional myosin-Ic	Nuclear bodies; Plasma membrane (Enhanced)
Q9Y6N5|SQOR_HUMAN	437.39	77	67	67	SQOR	Sulfide:quinone oxidoreductase mitochondrial	Mitochondria (Supported)
P25705|ATPA_HUMAN	421.43	76	84	83	**ATP5F1A**	ATP synthase subunit alpha mitochondrial	Mitochondria (Supported)
tr|E9PDF6|E9PDF6_HUMAN	416.06	57	94	65	MYO1B	Unconventional myosin-Ib	Plasma membrane (Enhanced)
P02545|LMNA_HUMAN	393	76	76	74	LMNA	Prelamin-A/C	Nuclear speckles (Supported)
P15924|DESP_HUMAN	379.69	45	152	150	DSP	Desmoplakin	Cell Junctions (Supported)
Q86YZ3|HORN_HUMAN	376.92	19	58	58	HRNR	Hornerin	Mitochondria (Uncertain)
P40939|ECHA_HUMAN	368.45	67	50	49	**HADHA**	Trifunctional enzyme subunit alpha mitochondrial	Mitochondria (Enhanced)
P46940|IQGA1_HUMAN	355.78	40	69	66	IQGAP1	Ras GTPase-activating-like protein IQGAP1	Cell Junctions; Plasma membrane (Supported)
P11021|BIP_HUMAN	355.27	60	49	47	HSPA5	Endoplasmic reticulum chaperone BiP	Cytosol (Approved)
P35579|MYH9_HUMAN	352.35	43	83	73	MYH9	Myosin-9	Actin filaments; Plasma membrane (Supported); Additional: Cytosol; Nuclear bodies
Q9NZM1|MYOF_HUMAN	339.24	40	75	74	MYOF	Myoferlin	Vesicles (Supported); Additional: Centriolar satellite; Plasma membrane
P26038|MOES_HUMAN	331.23	72	59	45	MSN	Moesin	Plasma membrane (Enhanced)
P38646|GRP75_HUMAN	330.1	58	49	47	**HSPA9**	Stress-70 protein mitochondrial	Mitochondria (Supported)
Q9UJS0|CMC2_HUMAN	328.32	51	37	24	SLC25A13	Calcium-binding mitochondrial carrier protein Aralar2	Mitochondria (Supported)
P55084|ECHB_HUMAN	327.34	59	42	42	HADHB	Trifunctional enzyme subunit beta mitochondrial	Mitochondria (Supported)
P16144|ITB4_HUMAN	326.33	34	55	54	ITGB4	Integrin beta-4	Cell Junctions; Plasma membrane (Supported)
P51659|DHB4_HUMAN	325.28	56	37	37	HSD17B4	Peroxisomal multifunctional enzyme type 2	Peroxisomes (Enhanced)

Q16181|SEPT7_HUMAN	323.94	57	39	39	SEPTIN7	Septin-7	Actin filaments; Cytosol (Supported); Additional: Midbody; Plasma membrane
P06733|ENOA_HUMAN	322.24	71	33	30	ENO1	Alpha-enolase	Cytosol; Plasma membrane (Enhanced)
P11142|HSP7C_HUMAN	321.72	52	39	23	**HSPA8**	Heat shock cognate 71 kDa protein	Nucleoplasm (Approved); Additional: Vesicles
P22695|QCR2_HUMAN	319.61	53	22	22	UQCRC2	Cytochrome b-c1 complex subunit 2 mitochondrial	Mitochondria (Enhanced)
P00533|EGFR_HUMAN	319.27	41	50	48	EGFR	Epidermal growth factor receptor	Cell Junctions; Plasma membrane (Supported); Additional: Nucleoli; Nucleoplasm
P06576|ATPB_HUMAN	319.26	64	30	30	**ATP5F1B**	ATP synthase subunit beta mitochondrial	Mitochondria (Enhanced)
P38159|RBMX_HUMAN	319.26	69	41	16	RBMX	RNA-binding motif protein X chromosome	Nucleoplasm (Supported)
P04843|RPN1_HUMAN	317.93	58	42	42	**RPN1**	Dolichyl-diphosphooligosaccharide--protein glycosyltransferase subunit 1	Endoplasmic reticulum (Enhanced); Additional: Cytosol
Q9UHD8|SEPT9_HUMAN	317.78	65	44	44	SEPTIN9	Septin-9	Actin filaments (Enhanced)
Q5T9A4|ATD3B_HUMAN	316.92	60	44	9	ATAD3B	ATPase family AAA domain-containing protein 3B	Mitochondria (Enhanced)
P08670|VIME_HUMAN	315.99	76	51	40	**VIM**	Vimentin	Intermediate filaments (Supported)
Q09666|AHNK_HUMAN	313.95	21	99	99	AHNAK	Neuroblast differentiation-associated protein AHNAK	Cytosol; Plasma membrane (Enhanced)
P49411|EFTU_HUMAN	313.74	61	28	28	TUFM	Elongation factor Tu mitochondrial	Mitochondria (Enhanced)
Q8NBS9|TXND5_HUMAN	309.64	54	28	27	TXNDC5	Thioredoxin domain-containing protein 5	Endoplasmic reticulum (Approved)
tr|E7EQB2|E7EQB2_HUMAN	309.07	63	50	49	LTF	Lactotransferrin (Fragment)	
P31930|QCR1_HUMAN	307.66	62	28	27	UQCRC1	Cytochrome b-c1 complex subunit 1 mitochondrial	Mitochondria (Supported)
Q15459|SF3A1_HUMAN	306.27	41	36	36	SF3A1	Splicing factor 3A subunit 1	Nuclear speckles; Nucleoplasm (Enhanced)
Q12965|MYO1E_HUMAN	291.47	37	44	36	MYO1E	Unconventional myosin-Ie	Nucleoplasm; Plasma membrane (Approved); Additional: Cytosol

Q53H12|AGK_HUMAN	291.45	54	32	32	AGK	Acylglycerol kinase mitochondrial	Mitochondria (Supported); Additional: Vesicles
Q96E39|RMXL1_HUMAN	289.74	55	28	3	RBMXL1	RNA binding motif protein X-linked-like-1	Nucleoplasm (Approved)
P19367|HXK1_HUMAN	289.01	45	45	37	HK1	Hexokinase-1	Mitochondria (Supported)
tr|A0A0B4J2A4|A0A0B4J2A4_HUMAN	287.99	80	25	25	ACAA2	3-ketoacyl-CoA thiolase mitochondrial	Mitochondria (Supported)
Q13835|PKP1_HUMAN	287.23	45	28	28	PKP1	Plakophilin-1	Nucleoplasm; Plasma membrane (Approved)
P14923|PLAK_HUMAN	286.55	52	44	36	JUP	Junction plakoglobin	Cell Junctions; Plasma membrane (Supported); Additional: Vesicles
Q86VI3|IQGA3_HUMAN	285.28	30	47	44	IQGAP3	Ras GTPase-activating-like protein IQGAP3	Nucleoli rim (Approved); Additional: Mitotic chromosome; Nucleoplasm
P20700|LMNB1_HUMAN	285.19	57	49	41	**LMNB1**	Lamin-B1	Nuclear membrane (Supported)
Q00839|HNRPU_HUMAN	283.3	36	36	36	HNRNPU	Heterogeneous nuclear ribonucleoprotein U	Nucleoplasm (Supported)
P31040|SDHA_HUMAN	283.2	44	25	25	**SDHA**	Succinate dehydrogenase [ubiquinone] flavoprotein subunit mitochondrial	Mitochondria (Supported); Additional: Nucleoli
tr|A0A0C4DGS1|A0A0C4DGS1_HUMAN	282.59	35	17	17	**DDOST**	Dolichyl-diphosphooligosaccharide--protein glycosyltransferase 48 kDa subunit	Endoplasmic reticulum (Supported)
P0DMV8|HS71A_HUMAN	281.18	40	31	13	**HSPA1A**	Heat shock 70 kDa protein 1A	Nucleoplasm; Vesicles (Approved); Additional: Cytosol
P00367|DHE3_HUMAN	280.31	54	28	28	**GLUD1**	Glutamate dehydrogenase 1 mitochondrial	Mitochondria (Supported)

Note ***Genes in green** font denoted the proteins identified as E-protein interactors in reference [2]. Genes in red highlighted the proteins denoted to be (partially) distributed on plasma membrane.

Table S4. List of the proteins pulled-down by EC_{38} peptides and annotated as “plasma membrane” in “subcellular location” (PM112) plus ATP5A/B

Accession	-10lgP	Coverage (%)	#Peptides	#Unique	GeneName	Description
O00159|MYO1C_HUMAN	445.48	61	99	94	MYO1C	Unconventional myosin-Ic
tr|E9PDF6|E9PDF6_HUMAN	416.06	57	94	65	MYO1B	Unconventional myosin-Ib
P46940|IQGA1_HUMAN	355.78	40	69	66	IQGAP1	Ras GTPase-activating-like protein IQGAP1
P35579|MYH9_HUMAN	352.35	43	83	73	MYH9	Myosin-9
Q9NZM1|MYOF_HUMAN	339.24	40	75	74	MYOF	Myoferlin
P26038|MOES_HUMAN	331.23	72	59	45	MSN	Moesin
P16144|ITB4_HUMAN	326.33	34	55	54	ITGB4	Integrin beta-4
Q16181|SEPT7_HUMAN	323.94	57	39	39	SEPTIN7	Septin-7
P06733|ENOA_HUMAN	322.24	71	33	30	ENO1	Alpha-enolase
P00533|EGFR_HUMAN	319.27	41	50	48	EGFR	Epidermal growth factor receptor
Q09666|AHNK_HUMAN	313.95	21	99	99	AHNAK	Neuroblast differentiation-associated protein AHNAK
Q12965|MYO1E_HUMAN	291.47	37	44	36	MYO1E	Unconventional myosin-Ie
Q13835|PKP1_HUMAN	287.23	45	28	28	PKP1	Plakophilin-1
P14923|PLAK_HUMAN	286.55	52	44	36	JUP	Junction plakoglobin
Q9BTU6|P4K2A_HUMAN	269.6	59	30	30	PI4K2A	Phosphatidylinositol 4-kinase type 2-alpha
O75955|FLOT1_HUMAN	257.33	57	25	25	FLOT1	Flotillin-1
P21333|FLNA_HUMAN	249.95	18	35	35	FLNA	Filamin-A
O43491|E41L2_HUMAN	248.9	31	30	30	EPB41L2	Band 4.1-like protein 2
P09543|CN37_HUMAN	248.59	42	27	27	CNP	2' 3'-cyclic-nucleotide 3'-phosphodiesterase
P29317|EPHA2_HUMAN	237.51	28	24	23	**EPHA2***	Ephrin type-A receptor 2
P50552|VASP_HUMAN	235.95	51	18	18	VASP	Vasodilator-stimulated phosphoprotein
tr|B5MCX3|B5MCX3_HUMAN	235.8	55	20	19	SEPTIN2	Septin 2
tr|E7EQR4|E7EQR4_HUMAN	230.29	34	23	11	EZR	Ezrin
P07355|ANXA2_HUMAN	229.63	54	23	23	ANXA2	Annexin A2
tr|C9JZR2|C9JZR2_HUMAN	229.53	34	29	29	CTNND1	Catenin delta-1
tr|A0A087WY71|A0A087WY71_HUMAN	226.52	51	27	27	**AP2M1**	AP-2 complex subunit mu
tr|A0A2R8Y7M3|A0A2R8Y7M3_HUMAN	222.54	32	20	5	RDX	Radixin
Q96RT1|ERBIN_HUMAN	221.8	24	27	27	ERBIN	Erbin
Q9ULF5|S3 (45-50kd)9AA_HUMAN	217.86	18	20	20	**SLC39A10**	Zinc transporter ZIP10

tr|F5GZS6|F5GZS6_HUMAN	216.61	31	17	17	SLC3A2	4F2 cell-surface antigen heavy chain
Q14254|FLOT2_HUMAN	215.71	42	20	20	FLOT2	Flotillin-2
P05556|ITB1_HUMAN	215.71	27	21	21	**ITGB1**	Integrin beta-1
Q16658|FSCN1_HUMAN	211.27	42	19	19	**FSCN1**	Fascin
P06702|S10A9_HUMAN	210.19	68	14	14	S100A9	Protein S100-A9
P67809|YBOX1_HUMAN	207.83	36	12	12	YBX1	Nuclease-sensitive element-binding protein 1
O94851|MICA2_HUMAN	201.62	18	20	20	**MICAL2**	[F-actin]-monooxygenase MICAL2
P35221|CTNA1_HUMAN	199.89	22	16	16	CTNNA1	Catenin alpha-1
tr|A0A0C4DFT3|A0A0C4DFT3_HUMAN	198.98	25	17	17	DLG1	Disks large homolog 1
P04406|G3P_HUMAN	196.82	54	14	14	GAPDH	Glyceraldehyde-3-phosphate dehydrogenase
Q8TEW0|PARD3_HUMAN	195.4	22	27	27	PARD3	Partitioning defective 3 homolog
Q9Y305|ACOT9_HUMAN	193.72	33	15	15	**ACOT9**	Acyl-coenzyme A thioesterase 9 mitochondrial
P35240|MERL_HUMAN	191.5	32	19	18	NF2	Merlin
P29508|SPB3_HUMAN	189.09	45	16	7	SERPINB3	Serpin B3
tr|H0Y512|H0Y512_HUMAN	188.46	44	18	18	APMAP	Adipocyte plasma membrane-associated protein (Fragment)
tr|H0Y2Y8|H0Y2Y8_HUMAN	183.61	32	13	13	ZYX	Zyxin (Fragment)
tr|A0A494C0G5|A0A494C0G5_HUMAN	183.02	10	15	15	AGRN	Agrin
P11047|LAMC1_HUMAN	181.88	11	14	14	LAMC1	Laminin subunit gamma-1
tr|A0A3B3ITE1|A0A3B3ITE1_HUMAN	181.04	13	14	13	TJP2	Tight junction protein ZO-2 (Fragment)
P43121|MUC18_HUMAN	180.57	26	13	13	MCAM	Cell surface glycoprotein MUC18
P04083|ANXA1_HUMAN	180.55	29	10	10	ANXA1	Annexin A1
P13639|EF2_HUMAN	176.8	22	14	14	EEF2	Elongation factor 2
P22735|TGM1_HUMAN	171.56	18	15	15	TGM1	Protein-glutamine gamma-glutamyltransferase K
tr|A0A0G2JNZ2|A0A0G2JNZ2_HUMAN	167.97	16	24	24	SCRIB	Protein scribble homolog
Q9C0B5|ZDHC5_HUMAN	166.26	30	16	16	ZDHHC5	Palmitoyltransferase ZDHHC5
Q8IVF7|FMNL3_HUMAN	165.39	14	13	8	FMNL3	Formin-like protein 3
O60292|SI1L3_HUMAN	164.04	12	19	19	SIPA1L3	Signal-induced proliferation-associated 1-like protein 3
P05362|ICAM1_HUMAN	160.34	18	8	8	ICAM1	Intercellular adhesion molecule 1
P16070|CD44_HUMAN	159.8	14	8	8	CD44	CD44 antigen
P21589|5NTD_HUMAN	159.73	18	10	10	NT5E	5'-nucleotidase
P29966|MARCS_HUMAN	158.38	34	7	7	MARCKS	Myristoylated alanine-rich C-kinase substrate

Q16513|PKN2_HUMAN	154.93	17	15	15	PKN2	Serine/threonine-protein kinase N2
Q15139|KPCD1_HUMAN	153.01	13	10	6	PRKD1	Serine/threonine-protein kinase D1
Q01469|FABP5_HUMAN	152.06	40	9	9	FABP5	Fatty acid-binding protein 5
O75146|HIP1R_HUMAN	151.8	12	11	10	HIP1R	Huntingtin-interacting protein 1-related protein
Q9Y490|TLN1_HUMAN	151.24	7	13	13	TLN1	Talin-1
O96013|PAK4_HUMAN	151.21	20	10	10	PAK4	Serine/threonine-protein kinase PAK 4
Q96PD2|DCBD2_HUMAN	150.35	14	9	9	DCBLD2	Discoidin CUB and LCCL domain-containing protein 2
P62987|RL40_HUMAN	148.68	37	6	6	**UBA52**	Ubiquitin-60S ribosomal protein L40
Q86XL3|ANKL2_HUMAN	147.44	16	15	15	**ANKLE2**	Ankyrin repeat and LEM domain-containing protein 2
tr|A0A0G2JI36|A0A0G2JI36_HUMAN	144.53	33	7	5	HLA-A	HLA class I histocompatibility antigen A-3 alpha chain
tr|E7EN95|E7EN95_HUMAN	143.82	5	10	10	FLNB	Filamin-B
Q96HC4|PDLI5_HUMAN	142.31	19	9	9	PDLIM5	PDZ and LIM domain protein 5
tr|H7C3C4|H7C3C4_HUMAN	141.83	12	8	3	SLC4A7	Anion exchange protein (Fragment)
tr|B4DUR8|B4DUR8_HUMAN	138.15	20	10	10	CCT3	T-complex protein 1 subunit gamma
Q99816|TS101_HUMAN	136.52	21	7	7	TSG101	Tumor susceptibility gene 101 protein
Q16643|DREB_HUMAN	134	14	9	9	DBN1	Drebrin
tr|A0A0G2JKZ1|A0A0G2JKZ1_HUMAN	133.9	15	6	6	TAPBP	Tapasin
Q14574|DSC3_HUMAN	132.42	10	8	7	DSC3	Desmocollin-3
P27816|MAP4_HUMAN	132.13	9	8	8	MAP4	Microtubule-associated protein 4
Q9NZT1|CALL5_HUMAN	131.96	32	4	4	CALML5	Calmodulin-like protein 5
Q6YHK3|CD109_HUMAN	130.4	11	12	12	CD109	CD109 antigen
A1X283|SPD2B_HUMAN	130.23	12	10	10	SH3PXD2B	SH3 and PX domain-containing protein 2B
Q9Y6M5|ZNT1_HUMAN	129.19	24	10	10	SLC30A1	Zinc transporter 1
Q9Y4D1|DAAM1_HUMAN	128.71	8	8	8	DAAM1	Disheveled-associated activator of morphogenesis 1
Q13433|S3 (45-50kd)9A6_HUMAN	128.36	8	6	6	SLC39A6	Zinc transporter ZIP6
O95425|SVIL_HUMAN	126.33	5	8	8	SVIL	Supervillin
P26006|ITA3_HUMAN	125.84	6	6	6	**ITGA3**	Integrin alpha-3
Q9UHB6|LIMA1_HUMAN	125.59	14	10	10	LIMA1	LIM domain and actin-binding protein 1
P63092|GNAS2 (105-120kd)_HUMAN	125.14	16	6	4	GNAS	Guanine nucleotide-binding protein G(s) subunit alpha isoforms short

Q96I99|SUCB2_HUMAN	121.18	25	9	9	SUCLG2	Succinate--CoA ligase [GDP-forming] subunit beta mitochondrial
tr|E9PFQ4|E9PFQ4_HUMAN	120.74	8	8	8	PHLDB2	Pleckstrin homology-like domain family B member 2 (Fragment)
tr|A0A0U1RQF0|A0A0U1RQF0_HUMAN	119.9	4	7	7	FASN	Fatty acid synthase
Q6NYC8|PPR18_HUMAN	117.91	14	7	7	PPP1R18	Phostensin
Q16555|DPYL2_HUMAN	116.2	15	7	4	DPYSL2	Dihydropyrimidinase-related protein 2
P20020|AT2B1_HUMAN	114.32	7	6	6	**ATP2B1**	Plasma membrane calcium-transporting ATPase 1
P04792|HSPB1_HUMAN	113.82	38	5	5	HSPB1	Heat shock protein beta-1
P13796|PLSL_HUMAN	113.31	16	8	8	LCP1	Plastin-2
Q9NZN4|EHD2_HUMAN	112.82	11	6	6	EHD2	EH domain-containing protein 2
P31431|SDC4_HUMAN	112.24	27	4	4	SDC4	Syndecan-4
tr|B4DTR1|B4DTR1_HUMAN	112.19	5	5	3	ERBB2	Receptor protein-tyrosine kinase
P27105|STOM_HUMAN	110.82	18	5	5	**STOM**	Erythrocyte band 7 integral membrane protein
tr|Q96II5|Q96II5_HUMAN	110.56	13	7	7	ARAF	ARAF protein
P80723|BASP1_HUMAN	109.34	46	6	6	BASP1	Brain acid soluble protein 1
tr|S4 (65-70kd)R3V8|S4 (65-70kd)R3V8_HUMAN	108.21	6	3	3	LSR	Lipolysis-stimulated lipoprotein receptor
tr|M0QXM4|M0QXM4_HUMAN	106.17	16	5	5	**SLC1A5**	Amino acid transporter
P12429|ANXA3_HUMAN	105.31	24	6	6	ANXA3	Annexin A3
tr|H9KV75|H9KV75_HUMAN	104.93	10	7	4	ACTN1	Alpha-actinin-1
O95297|MPZL1_HUMAN	104.86	13	3	3	MPZL1	Myelin protein zero-like protein 1
Q9HCY8|S10AE_HUMAN	104.18	39	4	4	S100A14	Protein S100-A14
Q5JTH9|RRP12_HUMAN	103.84	4	5	5	RRP12	RRP12-like protein
tr|C9J813|C9J813_HUMAN	103.64	15	6	6	CALD1	Caldesmon (Fragment)
tr|G3XAL9|G3XAL9_HUMAN	101.31	6	5	5	**SLC12A2**	Solute carrier family 12 (Sodium/potassium/chloride transporters) member 2 isoform CRA_a
P25705|ATPA_HUMAN	421.43	76	84	83	ATP5F1A	ATP synthase subunit alpha mitochondrial
P06576|ATPB_HUMAN	319.26	64	30	30	ATP5F1B	ATP synthase subunit beta mitochondrial

Note ***Green** font denoted the protein identified as E-protein interactor in reference [2].

REFERENCES

1. Zhang Y, Wang S, Wu Y, et al. Virus-Free and Live-Cell Visualizing SARS-CoV-2 Cell Entry for Studies of Neutralizing Antibodies and Compound Inhibitors. *Small Methods.* 2021;5(2):2001031.
2. Samavarchi-Tehrani P, Abdouni H, Knight J, et al. A SARS-CoV-2 – host proximity interactome. *bioRxiv 20200903282103.* 2020;doi: https://doi.org/10.1101/2020.09.03.282103.
